# Defective autophagy is a key feature of cerebral cavernous malformations

**DOI:** 10.15252/emmm.201505316

**Published:** 2015-09-28

**Authors:** Saverio Marchi, Mariangela Corricelli, Eliana Trapani, Luca Bravi, Alessandra Pittaro, Simona Delle Monache, Letizia Ferroni, Simone Patergnani, Sonia Missiroli, Luca Goitre, Lorenza Trabalzini, Alessandro Rimessi, Carlotta Giorgi, Barbara Zavan, Paola Cassoni, Elisabetta Dejana, Saverio Francesco Retta, Paolo Pinton

**Affiliations:** 1 Department of Morphology, Surgery and Experimental Medicine, Section of Pathology Oncology and Experimental Biology, University of FerraraFerrara, Italy; 2 Department of Clinical and Biological Sciences, University of TorinoTorino, Italy; 3 IFOM, FIRC Institute of Molecular OncologyMilano, Italy; 4 Department of Medical Sciences, University of TorinoTorino, Italy; 5 Department of Biotechnological and Applied Clinical Science, University of L’AquilaL’Aquila, Italy; 6 Department of Biomedical Sciences, University of PadovaPadova, Italy; 7 Department of Biotechnology, Chemistry and Pharmacy, University of SienaSiena, Italy

**Keywords:** autophagy, CCM, endothelial-to-mesenchymal transition (EndMt), mTOR, ROS

## Abstract

Cerebral cavernous malformation (CCM) is a major cerebrovascular disease affecting approximately 0.3–0.5% of the population and is characterized by enlarged and leaky capillaries that predispose to seizures, focal neurological deficits, and fatal intracerebral hemorrhages. Cerebral cavernous malformation is a genetic disease that may arise sporadically or be inherited as an autosomal dominant condition with incomplete penetrance and variable expressivity. Causative loss-of-function mutations have been identified in three genes, *KRIT1* (*CCM1*), *CCM2 (MGC4607),* and *PDCD10* (*CCM3*), which occur in both sporadic and familial forms. Autophagy is a bulk degradation process that maintains intracellular homeostasis and that plays essential quality control functions within the cell. Indeed, several studies have identified the association between dysregulated autophagy and different human diseases. Here, we show that the ablation of the *KRIT1* gene strongly suppresses autophagy, leading to the aberrant accumulation of the autophagy adaptor p62/SQSTM1, defective quality control systems, and increased intracellular stress. KRIT1 loss-of-function activates the mTOR-ULK1 pathway, which is a master regulator of autophagy, and treatment with mTOR inhibitors rescues some of the mole-cular and cellular phenotypes associated with CCM. Insufficient autophagy is also evident in *CCM2*-silenced human endothelial cells and in both cells and tissues from an endothelial-specific *CCM3*-knockout mouse model, as well as in human CCM lesions. Furthermore, defective autophagy is highly correlated to endothelial-to-mesenchymal transition, a crucial event that contributes to CCM progression. Taken together, our data point to a key role for defective autophagy in CCM disease pathogenesis, thus providing a novel framework for the development of new pharmacological strategies to prevent or reverse adverse clinical outcomes of CCM lesions.

## Introduction

Cerebral cavernous malformations (CCMs; OMIM 116860), which are also known as cavernous angiomas or cavernomas, are major vascular malformations consisting of closely clustered, abnormally dilated, and leaky capillary channels (caverns) lined by a thin endothelium and devoid of normal vessel structural components (Clatterbuck *et al*, 2001; Gault *et al*, 2004; Batra *et al*, 2009; Cavalcanti *et al*, 2012).

Cerebral cavernous malformation lesions are estimated to occur in 0.3–0.5% of the general population (Cavalcanti *et al*, 2012) and can either remain clinically silent or cause serious clinical symptoms, such as headaches, neurological deficits, seizures, strokes, and intracerebral hemorrhages (Gault *et al*, 2004; Batra *et al*, 2009). Approximately 30% of people with CCM lesions will eventually develop clinical symptoms.

Cerebral cavernous malformation has a known genetic origin and may either arise sporadically or be inherited as an autosomal dominant condition with incomplete penetrance and variable expressivity. Genetic studies have identified three genes whose loss-of-function mutations cause CCM: *KRIT1 (CCM1), MGC4607 (CCM2), and PDCD10* (*CCM3*), which account for approximately 50, 20, and 10% of CCM cases, respectively. The remaining 20% of cases have been attributed to mutations in a fourth unidentified CCM gene (Riant *et al*, 2010). Notably, the hereditary form of this illness is often associated with multiple cavernous angiomas, whereas the sporadic form typically presents as a solitary lesion.

At present, no direct therapeutic approaches for CCM disease exist other than the surgical removal of accessible lesions in patients with recurrent hemorrhage or intractable seizures. In particular, novel pharmacological strategies are required for preventing the *de novo* formation of CCM lesions in susceptible individuals and the progression of the disease. Useful insights into the definition of novel approaches for CCM disease prevention and treatment could be derived from a deep understanding of the mechanisms underlying CCM pathogenesis.

Macroautophagy (termed autophagy in this manuscript) is a bulk degradation process that occurs in two primary steps: (i) the sequestration of proteins and organelles into double-membrane vesicles called autophagosomes and (ii) their subsequent degradation through the fusion of autophagosomes with lysosomes (Xie & Klionsky, 2007; Feng *et al*, 2014). By selectively degrading harmful protein aggregates or damaged organelles, autophagy maintains intracellular homeostasis and plays essential quality control functions within the cell (Mizushima & Komatsu, 2011).

Defective autophagy occurs in several pathological conditions, including cancers, neurodegenerative and cardiovascular diseases, and metabolic disorders (Levine & Kroemer, 2008; Choi *et al*, 2013). The suppression of autophagy causes the accumulation of proteins and potentially hazardous intracellular structures, thereby inducing high levels of metabolic stress and limiting organelle functionality. Consequently, using a pharmacological approach to re-establish physiological levels of autophagy may be beneficial in treating certain diseases. Nevertheless, several clinical trials are currently based on the employment of agents acting on autophagy induction (Choi *et al*, 2013; Jiang & Mizushima, 2014).

In the present study, we show that human CCM lesions display increased levels of p62/SQSTM1, an autophagic marker that accumulates when autophagy is inhibited, and demonstrate that both KRIT1 and CCM3 loss-of-function impair autophagy through the up-regulation of the mechanistic target of rapamycin (mTOR) pathway, leading to a defective quality control system and the accumulation of aberrant and aggregated proteins. Our data raise the possibility that therapeutic activation of autophagy might prevent or reverse adverse clinical outcomes, thus improving the long-term prognosis of CCM patients.

## Results and Discussion

### KRIT1 deletion suppresses autophagy

To study the contribution of autophagy to CCM pathogenesis, we investigated whether KRIT1 down-regulation would lead to the impairment of autophagy in endothelial cell lines.

Endothelial-specific KRIT1 knockout (KO) in mice produced lesions that were identical to the CCM malformations observed in humans (Boulday *et al*, 2011; Maddaluno *et al*, 2013). We used KRIT1-KO lung endothelial cells derived from *KRIT1*^fl/fl^ mice treated with Tat-Cre recombinase (Maddaluno *et al*, 2013).

**Figure 1:**
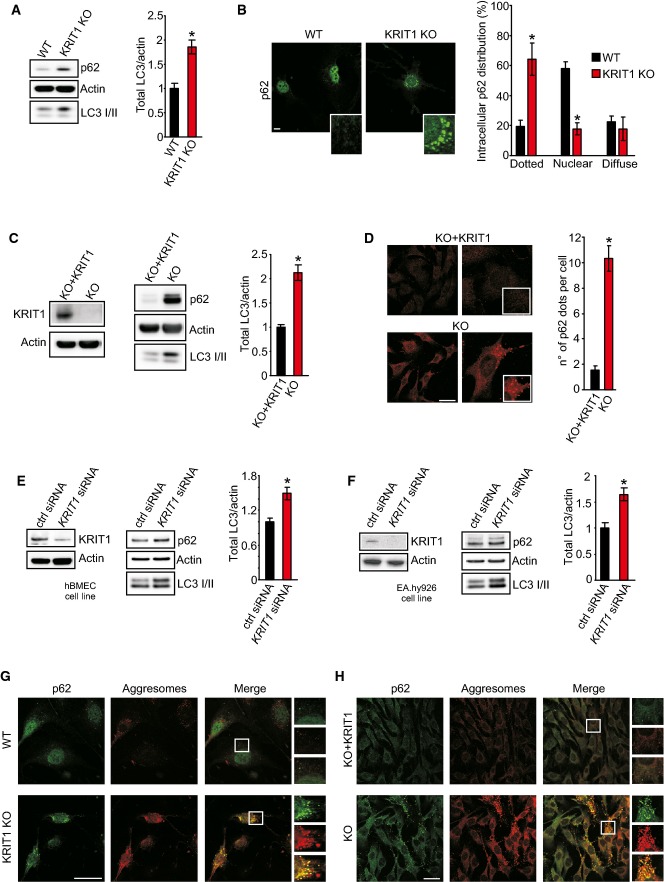
KRIT1-ablated cells display autophagy suppression Immunoblot analysis of p62 and LC3 I/II in KRIT1 wt and KRIT1-KO endothelial cells. Actin was used as a loading control. Quantification of total LC3 on actin is reported (*P =* *0.02712). The results are representative of three independent experiments.

p62/SQSTM1 acts as a receptor for ubiquitinated cargoes and delivers them to the autophagosome, and p62 itself is incorporated into the autophagosome and subsequently degraded by autophagy (Komatsu *et al*, 2007). The autophagy protein microtubule-associated protein 1 light chain 3 (LC3) is present in the cytosol in the LC3-I form, until it is modified to a cleaved and lipidated membrane-bound form (LC3-II), which is localized to autophagosomes. Thus, in addition to p62 accumulation, another typical trait of autophagy inhibition consists of increased amounts of the cytosolic non-lipidated form of LC3 (LC3-I) and of total LC3 (Mizushima *et al*, 2010; Wang *et al*, 2012). As shown in Fig1A, KRIT1 deficiency was associated with defective autophagy, displaying increased levels of p62 and total LC3.

Representative images of p62 dots in KRIT1 wt and KRIT1-KO endothelial cells. Scale bar, 20 μm. Magnifications in insets. Right, quantitative analysis of p62 distribution of dots is reported (four independent experiments; *n* = 35 cells per group). *P =* *0.00542 (dotted); *P =* *0.00014 (nuclear).

Immunoblot analysis of p62 and LC3 I/II in KRIT1-KO and KRIT1-KO re-expressing KRIT1 (KO+KRIT1) MEFs. Left, immunoblot showing KRIT1 levels in KRIT1-KO and KO+KRIT1 cells. Right, immunoblots for p62 and LC3 I/II. Actin was used as a loading marker. Quantification of total LC3 on actin is reported (*P =* *0.01248). The results are representative of three independent experiments.

Representative images of p62 dots in KO+KRIT1 (top) and KRIT1-KO cells (bottom). Scale bar, 50 μm. Magnifications in insets. Right, quantitative analysis of the number of p62 dots per cell is shown (four independent experiments; *n* = 50 cells per group). *P =* *7.18e^−14^.

Immunoblot analysis of hBMECs transiently transfected with control siRNA or *KRIT1* siRNA. Left, evaluation of siRNA efficiency with antibody directed against KRIT1. Right, immunoblots for p62 and LC3 I/II. Actin was used as a loading marker. Quantification of total LC3 on actin is reported (*P =* *0.03071). The results are representative of three independent experiments.

Immunoblot analysis of EA.hy926 cells transiently transfected with control siRNA or *KRIT1* siRNA. Left, evaluation of siRNA efficiency with antibody directed against KRIT1. Right, immunoblot for p62 and LC3 I/II. Actin was used as a loading marker. Quantification of total LC3 on actin is reported (*P =* *0.02527). The results are representative of three independent experiments.

Immunofluorescence analysis of p62 (green) and ProteoStat Aggresome staining detection reagent (red) in KRIT1 wt and KRIT1-KO lung endothelial cells. The yellow signal in the merged images represents an overlapping spatial relationship between green and red fluorescence. Magnification in insets. Scale bar, 50 μm. The images are representative of four independent experiments.

Immunofluorescence analysis of p62 (green) and ProteoStat Aggresome staining detection reagent (red) in KRIT1-KO re-expressing KRIT1 (KO+KRIT1) and KRIT1-KO MEFs. The yellow signal in the merged images represents an overlapping spatial relationship between green and red fluorescence. Magnification in insets. Scale bar, 50 μm. The images are representative of four independent experiments. Source data are available online for this figure.

Upon autophagy inhibition, p62 has been reported to be present in several types of cytoplasmic inclusions and to display a typical punctate pattern (Bjorkoy *et al*, 2005). Importantly, analysis of p62 distribution through immunofluorescence staining revealed a nuclear-enriched pattern with rare cytoplasmic dots in ∼60% of KRIT1 wild-type (wt) cells. Conversely, in KO endothelial cells, the protein is primarily cytoplasmatic, forming intense perinuclear bodies with weak staining in the nucleus (Fig1B).

To investigate whether defective autophagy in CCM is a cell-autonomous process, we took advantage of *KRIT1* KO (KRIT1-KO) mouse embryonic fibroblasts (MEFs), a previously established and characterized cellular model that allowed the identification of new molecules and mechanisms involved in CCM pathogenesis (Goitre *et al*, 2010, 2014), providing novel therapeutic perspectives (Gibson *et al*, 2015; Moglia *et al*, 2015). Compared with KRIT1-KO MEFs re-expressing KRIT1 (Fig1C; KO+KRIT1), KRIT1-KO MEFs (Fig1C; KO) displayed increased levels of p62 as well as significantly increased levels of total LC3 protein (Fig1C). Moreover, immunostaining analysis revealed that KRIT1 depletion led to increases in the number of p62-containing bodies (Fig1D), with diameters of approximately 1.5 μm.

Next, we examined whether KRIT1 ablation also inhibits autophagy in human cells. The silencing of KRIT1 suppressed autophagy in both the human cerebral microvascular endothelial cell line hBMEC (Fig1E) and the human umbilical vein cell line EA.hy926 (Fig1F), as evidenced by increased p62 and LC3 accumulation.

p62 protein expression is highly regulated at the transcriptional level via the JNK pathway (Puissant *et al*, 2010) or the NRF2 transcription factor, particularly under oxidative stress (He & Klionsky, 2009; Puissant *et al*, 2012). Considering that KRIT1 is involved in reactive oxygen species (ROS) homeostasis (Goitre *et al*, 2010, 2014; Jung *et al*, 2014), we tested whether p62 accumulation in KRIT1-KO cells was associated with autophagy inhibition rather than with ROS-dependent transcriptional effects. As expected, treatment with the antioxidant N-acetylcysteine (NAC) decreased p62 levels, but the disruption of *KRIT1* still induced p62 accumulation (Appendix Fig S1A). Moreover, similar results were obtained using the protein synthesis inhibitor cycloheximide (CHX) (Appendix Fig S1B), further supporting the notion that the inhibition of autophagy-dependent protein turnover upon KRIT1 loss contributes to p62 accumulation. Consistently, no differences in *p62* mRNA levels between wt and KRIT1-KO endothelial cells have been detected (Appendix Fig S1C). Importantly, when autophagy-mediated degradation is inhibited, p62 appears to be partially detergent insoluble (Klionsky *et al*, 2012); therefore, the lysates were divided between Triton X-100 (TX-100)-soluble and TX-100-insoluble fractions and subsequently analyzed for their protein content. The loss of KRIT1 in both endothelial cells (Appendix Fig S1D) and MEFs (Appendix Fig S1E) promoted increased levels of p62 in both the soluble and insoluble fractions, which is consistent with previous observations made under defective autophagy and high protein aggregation conditions (Waguri & Komatsu, 2009; Fujita *et al*, 2011; Magnaudeix *et al*, 2013).

Autophagy is responsible for the degradation of large structures such as organelles and protein aggregates (Rabinowitz & White, 2010; Cheng *et al*, 2013). Consequently, we analyzed whether the defective autophagy observed upon *KRIT1* loss might induce the accumulation of aggresome-like structures. As shown in Fig1G, we observed greater colocalization between p62 and aggresomes in endothelial KRIT1-KO cells, as well as extremely high fluorescence intensity of aggresome-like inclusion bodies. The same results have been obtained in different cellular systems, such as MEFs (Fig1H) or *KRIT1*-silenced hBMECs and EA.hy926 cells (Appendix Fig S1F and G), indicating that the loss of KRIT1 promotes the accumulation of aberrant proteins that could be reasonably ascribed to defective autophagy. Virtually identical observations have been reported for other autophagy-deficient scenarios (Maejima *et al*, 2013; Wolf *et al*, 2013).

These findings suggest that KRIT1 ablation is sufficient to suppress autophagy in a cell-autonomous manner. Indeed, KRIT1 silencing or disruption in four different cellular contexts has been shown to result in the expression of typical markers of defective autophagy, such as increased accumulation of p62 and increased amounts of LC3-I and of total LC3.

### KRIT1 deletion induces up-regulation of the mTOR-ULK1 pathway

The mTOR signaling network is recognized as the most important regulator of autophagy, and its implication in a wide range of diseases has been largely documented (Laplante & Sabatini, 2012). Direct selective inhibition of mTOR, through the allosteric inhibitor rapamycin or the small molecule ATP-competitive inhibitor Torin1, induces autophagy in many cell types (Kundu, 2011). Consequently, we tested whether the defective autophagy observed upon *KRIT1* deletion resulted from dysregulation of the mTOR pathway.

**Figure 2:**
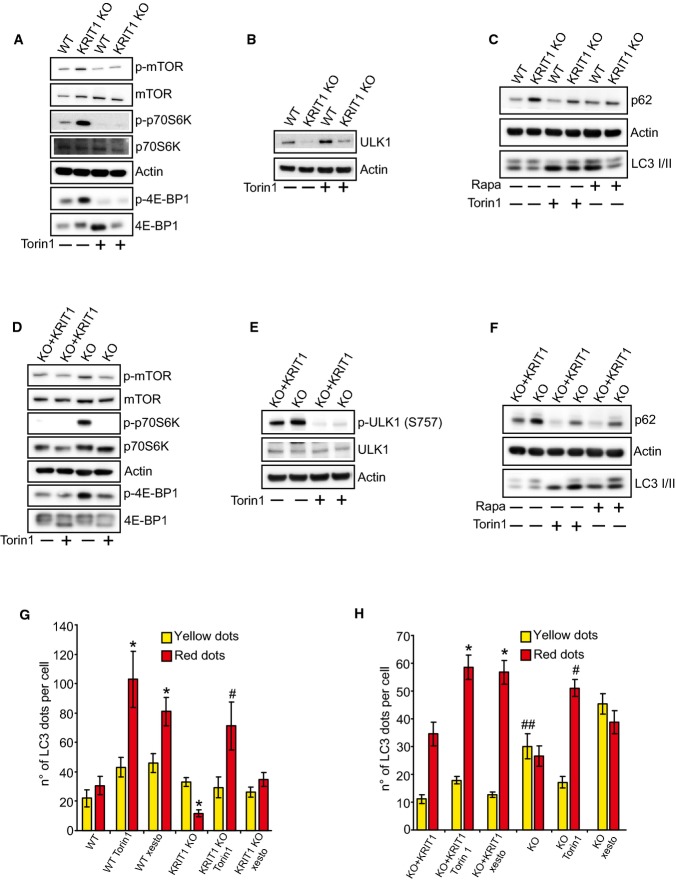
KRIT1 loss-of-function activates the mTOR-ULK1 pathway Immunoblot analysis with antibodies directed against phosphorylated mTOR (Ser 2448), total mTOR, phosphorylated p70 S6 Kinase (Ser 371), total p70 S6 Kinase, phosphorylated 4E-BP1 (Thr 37/46), and total 4E-BP1; actin was used as a loading marker. Where indicated, KRIT1 wt and KRIT1-KO endothelial cells were treated with 100 nM Torin1 for 4 h. The results are representative of three independent experiments.

Immunoblot analysis revealed marked up-regulation of mTOR signaling in KRIT1-KO endothelial cells, as evidenced by the increased phosphorylation of both mTOR and its downstream targets p70S6k and 4E-BP1 (Fig2A). Importantly, treatment with Torin1 suppressed mTOR activation even in KO cells, suggesting that a pharmacological approach based on mTOR inhibition might re-activate autophagy in these cells.

Immunoblot analysis of total ULK1 and actin in KRIT1 wt and KRIT1-KO endothelial cells. Where indicated, cells were treated with 100 nM Torin1 for 4 h. The results are representative of three independent experiments.

Immunoblot analysis of p62, LC3 I/II, and actin in KRIT1 wt and KRIT1-KO endothelial cells treated with 100 nM Torin1 or 500 nM rapamycin for 4 h. The results are representative of three independent experiments.

Immunoblot analysis with antibodies directed against phosphorylated mTOR (Ser 2448), total mTOR, phosphorylated p70 S6 Kinase (Ser 371), total p70 S6 Kinase, phosphorylated 4E-BP1 (Thr 37/46), and total 4E-BP1; actin was used as a loading marker. Where indicated, KRIT1-KO re-expressing KRIT1 (KO+KRIT1) and KRIT1-KO MEFs were treated with 100 nM Torin1 for 4 h. The results are representative of three independent experiments.

Immunoblot analysis of phosphorylated ULK1 (Ser 757), total ULK1, and actin in KRIT1 KO+KRIT1, and KRIT1 KO MEFs. Where indicated, cells were treated with 100 nM Torin1 for 4 h. The results are representative of three independent experiments.

Immunoblot analysis of p62, actin, LC3 I/II in KO+KRIT1 and KRIT1-KO cells. Where indicated, cells were treated with 100 nM Torin1 for 4 h or 500 nM rapamycin for 4 h. The results are representative of three independent experiments.

KRIT1 wt and KRIT1-KO endothelial cells were transiently transfected with mRFP-GFP-LC3. Where indicated, the cells were treated with 100 nM Torin1 for 4 h or 2 μM xestospongin B for 4 h. The differences in the autophagic flux were evaluated by counting the yellow LC3 I/II dots/cell (RFP^+^GFP^+^) and red LC3 dots/cell (RFP^+^GFP^−^) for each condition. Yellow dots: autophagosomes; red dots: autophagolysosomes. *P =* *5.74e^−5^ (red dots, WT ctrl vs. WT Tor1); *P =* *9.62e^−5^ (red dots, WT ctrl vs. WT xesto); *P =* *0.00727 (red dots, WT ctrl vs. KO ctrl); ^#^*P *=* *0.00046 (red dots, KO ctrl vs. KO Tor1). The data are expressed as the mean ± s.e.m.

KO+KRIT1 and KRIT1-KO MEFs were transiently transfected with the mRFP-GFP-LC3 tandem construct. Where indicated, the cells were treated with 100 nM Torin1 for 4 h or 2 μM xestospongin B for 4 h. The differences in the autophagic flux were evaluated by counting the yellow LC3 I/II dots/cell (RFP^+^GFP^+^) and red LC3 dots/cell (RFP^+^GFP^−^) for each condition. Yellow dots: autophagosomes; red dots: autophagolysosomes. *P =* *0.00023 (red dots, KO+KRIT1 ctrl vs. KO+KRIT1 Tor1); *P =* *0.00045 (red dots, KO+KRIT1 ctrl vs. KO+KRIT1 xesto); ^#^*P *=* *3.08e^−6^ (red dots, KO ctrl vs. KO Tor1); ^##^*P *=* *6.73e^−5^ (yellow dots, KO+KRIT1 ctrl vs. KO ctrl). The data are expressed as the mean ± s.e.m. of four independent experiments. Source data are available online for this figure.

Among the different targets of mTOR, ULK1, the mammalian homolog of yeast ATG1, is deeply involved in the regulation of autophagy through its interactions with several autophagy-related proteins (Wong *et al*, 2013). For example, ULK1-deficient mice display suppressed autophagy (Hara *et al*, 2008; Kundu *et al*, 2008). mTOR phosphorylates ULK1 at Ser 757 to inhibit autophagy (Kim *et al*, 2011). Notably, mTOR exerts a further restriction on autophagy by indirectly inhibiting ULK1 activity and stability (Nazio *et al*, 2013).

In our study, endothelial KRIT1 ablation significantly decreased the baseline levels of ULK1 and inhibition of mTOR by Torin1 treatment increased the total amounts of ULK1 protein (Fig2B), indicating that reduced ULK1 levels in KRIT1-KO endothelial cells might be dependent on higher mTOR activity. Indeed, impaired ULK1 stabilization and activity occur when the autophagy regulator AMBRA1 is highly phosphorylated by mTOR kinase at position 52 (Nazio *et al*, 2013). As shown in Appendix Fig S2A, AMBRA1 phosphorylation at Serine 52 is more abundant upon KRIT1 deletion compared to wt endothelial cells.

Then, we tested the efficacy of mTOR inhibition for reinstating autophagy under KRIT1 depletion. As evidenced by the increased LC3 I/II ratios and reduced p62 levels, both rapamycin and Torin1 effectively activated autophagy (Fig2C).

Next, we investigated mTOR activity in KRIT1-depleted MEFs. The autophagy defects observed in KRIT1-KO cells could be attributed to alterations of the mTOR-ULK1 pathway (Fig2D and E). In this case, we observed only a slight decrease in ULK1 expression in KRIT1-KO MEFs; however, mTOR-dependent phosphorylation of endogenous ULK1 at Ser 757 was increased (Fig2E and Appendix Fig S2B), indicating that mTOR might control ULK1 activity primarily by direct phosphorylation in this cellular context.

Interestingly, we observed a significant increase in the total amount of mTOR in both KRIT1-KO endothelial cells and fibroblasts (Fig2A and D and Appendix Fig S2B), which might reasonably affect the totality of active mTOR.

The use of rapamycin and mTOR kinase inhibitors significantly re-established autophagy (Fig2F). Importantly, the induction of autophagy was more robust in Torin1-treated cells, as evidenced by the greater inhibition of the mTOR pathway by Torin1 (Appendix Fig S2B). Consistently, Torin1, which is known to inhibit equally the two mTOR functional complexes (mTORC1 and mTORC2), has been reported to be more effective than rapamycin in inhibiting mTORC1, as well as to activate autophagy to a greater extent than rapamycin independently of its putative action on mTORC2 (Thoreen *et al*, 2009). Therefore, the efficacy of Torin1 treatment to drive autophagy even in KRIT1-KO cells might be likely attributable to its greater effect on autophagy, as compared with rapamycin.

One of the most useful methods for measuring autophagy is based on the mRFP-GFP-LC3 tandem construct assay (Mizushima *et al*, 2010). In cells expressing mRFP-GFP-LC3, the association of LC3 with autophagosomes can be visualized as yellow puncta due to the merge of green and red, whereas autolysosomes are detected as red puncta because the green fluorescence is quenched by the acidic pH of the lysosomal environment. Thus, if autophagic flux increases (i.e., upon pro-autophagic stimuli), the number of LC3 puncta increases, with a higher number of red puncta than the number of yellow puncta; conversely, when autophagic flux is impaired, only yellow puncta increase without a concomitant increase in red puncta.

Both Torin1 and xestospongin B, a mTOR-independent stimulus that induces autophagy by disrupting the molecular complex between inositol 1,4,5-trisphosphate receptor (IP3R) and Beclin-1 (Rubinsztein *et al*, 2012), activated autophagic flux in wt endothelial cells, whereas only Torin1 re-activated autophagy in KO cells (Fig2G). Similar results were obtained in KRIT1-KO MEFs (Fig2H). Notably, KO MEFs also displayed autophagy inhibition at the later stages of the process. This result might be related to the dual suppressive role played by mTOR, which inhibits autophagy not only at the initiation stage via suppression of the ULK1 complex but also at the degradation stage via inhibition of lysosomal function (Zhou *et al*, 2013a,b). Furthermore, the analysis of the lysosomal compartment through the transfection of GFP-tagged lysosomal-associated membrane protein (LAMP1-GFP) revealed the accumulation of clustered lysosomes in KO cells, displaying a morphological pattern similar to that of KRIT1-expressing cells that had been treated with the lysosomal inhibitor bafilomycin A1 (Appendix Fig S2C2013b).

KRIT1 loss-of-function leads to enhanced levels of intracellular ROS (Goitre *et al*, 2010; Jung *et al*, 2014) and cell proliferation (Maddaluno *et al*, 2013). Thus, we verified whether autophagy induction counteracts those KRIT1-dependent pathological processes. Measurements of hydrogen peroxide production using the ratiometric mitochondria-targeted HyPer probe (mt-HyPer) showed that Torin1 treatment of KO cells markedly reduced baseline ROS levels (Appendix Fig S3A). Importantly, the use of antioxidants such as NAC or Tempol did not affect the mTOR signaling over-activation observed in KRIT1-KO cells (Appendix Fig S3B); accordingly, ROS scavengers failed to trigger autophagy in KO cells (Appendix Fig S3C), suggesting that ROS accumulation is a consequence of mTOR activity and not *vice versa*. Furthermore, mTOR inhibitors strongly attenuated the proliferative rate of both KRIT1-KO endothelial cells and MEFs (Appendix Fig S3D and E).

Overall, these data suggest that KRIT1 loss inhibits autophagy through the up-regulation of the mTOR pathway and that the restoration of autophagy by mTOR inhibitors could significantly mitigate the metabolic disorders resulting from KRIT1 loss-of-function.

### Defective autophagy underlies major phenotypic signatures of CCM disease

**Figure 3:**
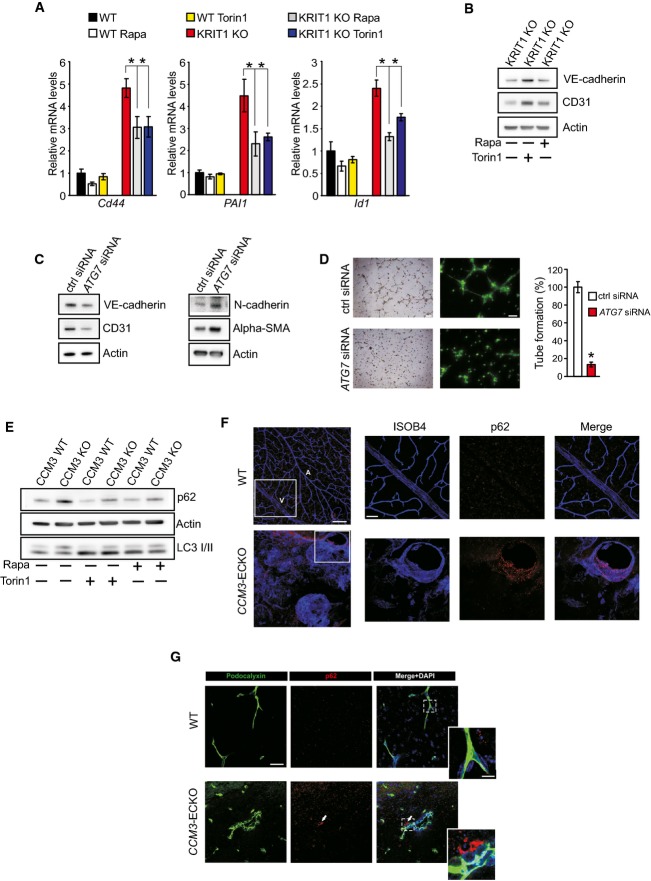
Defective autophagy underlies major phenotypic signatures of CCM disease *Cd44*, *PAI1* (also known as *Serpine1*), and *Id1* mRNA expression levels in KRIT1 wt and KRIT1-KO endothelial cells were assessed by quantitative real-time PCR. Where indicated, KRIT1 wt and KRIT1-KO endothelial cells were treated with 100 nM Torin1 or 500 nM rapamycin for 16h. The data are expressed as the mean ± s.e.m. *Cd44*: *P =* *0.02848 (KO ctrl vs. KO Rapa); *P =* *0.02605 (KO ctrl vs. KO Tor1). *PAI1*: *P =* *0.04446 (KO ctrl vs. KO Rapa); *P =* *0.03996 (KO ctrl vs. KO Tor1). *Id1*: *P =* *0.00266 (KO ctrl vs. KO Rapa); *P =* *0.01554 (KO ctrl vs. KO Tor1). *n* = 3 independent experiments.

To further clarify whether defective autophagy is involved in the pathogenesis of CCM, we investigated the relationship between autophagy and endothelial-to-mesenchymal transition (EndMt), a pathological signature that contributes to CCM progression (Maddaluno *et al*, 2013). KRIT1-KO endothelial cells displayed higher expression of typical markers that are associated with EndMt, such as *PAI1* (also known as *Serpine1*), *Cd44,* and *Id1* (Fig3A). Both Torin1 and rapamycin treatments inhibited the EndMt switch by lowering the expression of mesenchymal markers (Fig3A) and by increasing the levels of key endothelial markers such as CD31 (also known as Pecam-1) and vascular endothelial cadherin (VE-cadherin) (Fig3B).

Immunoblot analysis of CD31/Pecam-1, vascular endothelial cadherin (VE-cadherin), and actin in KRIT1-KO endothelial cells that were treated with 100 nM Torin1 or 500 nM rapamycin for 24 h. The results are representative of three independent experiments.

Immunoblot analysis of CD31/Pecam-1, vascular endothelial cadherin (VE-cadherin), N-cadherin, alpha-smooth muscle actin (alpha-SMA), and actin in HUVECs transfected with control siRNA or *ATG7* siRNA.

Formation of capillary-like structures measured by tube formation assays. HUVECs were transfected with control siRNA or *ATG7* siRNA for 72 h. Representative phase-contrast (Scale bar, 100 μm) and calcein-fluorescent (Scale bar, 50 μm) images were reported. All data are presented as percentage ± s.e.m from three different experiments performed in duplicate. *P =* *1.29e^−11^.

Immunoblot analysis of p62, LC3 I/II, and actin in CCM3 wt and CCM3-KO endothelial cells treated with 100 nM Torin1 or 500 nM rapamycin for 4 h. The results are representative of three independent experiments.

Representative immunostaining of retina sections from wt and a model of inducible and endothelial-specific CCM3-KO (*CCM3-*ECKO) at postnatal day 14. Endothelium was stained with isolectin B4 (ISOB4) (blue). A, artery; V, vein. p62 aggregates can be observed in endothelial cells forming retinal lesions in *CCM3-*ECKO animals (scale bar: 200 μm). Scale bar of magnifications: 100 μm.

Representative immunostaining of brain sections from wt and a model of inducible and endothelial-specific CCM3-knockout mice (*CCM3-*ECKO) at postnatal day 9. p62 aggregates can be observed in the proximity of CCM lesions (arrows). Cell nuclei (DAPI) are in blue. Scale bar, 30 μm. Smaller panel shows the magnifications of blood vessels (green). Scale bar, 10 μm. Source data are available online for this figure.

Down-regulation of the essential autophagy-related gene *ATG7* in human umbilical vein endothelial cells (HUVECs) suppressed autophagy (Appendix Fig S4A) and was associated with changes in the expression of markers of EndMt, such as a decrease in endothelial markers (CD31 and VE-cadherin) and a complementary increase in mesenchymal markers (N-cadherin and alpha-SMA; Fig3C). Moreover, *ATG7* silencing in HUVECs slowed the formation of capillary-like structures (Fig3D) but significantly increased the migratory capacity of these cells (Appendix Fig S4B). Importantly, inhibition of mTOR signaling reduced the migration of KRIT1-KO endothelial cells (Appendix Fig S4C and D). These data are consistent with recent observations (Zhang *et al*, 2013; Singh *et al*, 2015), translating the association between mTOR-dependent inhibition of autophagy and EndMt to CCM disease.

Intriguingly, a key role for p62 in the regulation of epithelial–mesenchymal transition has been recently reported (Bertrand *et al*, 2015), prompting us to investigate whether this concept could be extended to EndMt. Consistently, p62 down-regulation in KRIT1-ablated endothelial cells significantly lowered the expression of mesenchymal markers such as *PAI1*, *Cd44,* and *Id1* (Appendix Fig S4E), further supporting the existence of a significant correlation between EndMt and autophagy in CCM.

Because mutations in any of the three *CCM* genes lead to the onset of similar pathological signatures, the three CCM proteins likely share a common mechanism of action. Therefore, we examined the role of autophagy in CCM3-depleted endothelial cells derived from *Ccm3*^fl/fl^ mice (Bravi *et al*, 2015). Similar to KRIT1 down-regulation, we observed autophagy inhibition upon CCM3 ablation, which could be re-activated by treatment with mTOR inhibitors (Fig3E). Importantly, CCM3-KO endothelial cells displayed mTOR-ULK1 pathway up-regulation (Appendix Fig S4F).

To confirm the data observed *in vitro*, we analyzed whether autophagy inhibition also occurred *in vivo* upon CCM3 ablation. As in patients with CCM (Labauge *et al*, 2007), an inducible and endothelial-specific CCM3-KO mouse model (*CCM3-*ECKO) presented venous malformations at the periphery of the retinal vascular plexus. We found that p62 strongly accumulated in the endothelial cells that formed the vascular malformations (Fig3F and Appendix Fig S4G). Moreover, an analysis of murine CCM3-KO brain sections showed p62 clusters in the surrounding area of vascular malformations (Fig3G).

To complete the analysis of all three CCM genes, we investigated the effect of CCM2 down-regulation in human endothelial cells on autophagy. CCM2 silencing in EA.hy926 cells (Appendix Fig S5A) induced p62 accumulation, as well as increased levels of LC3-I and LC3-II (Appendix Fig S5B). Moreover, immunofluorescence staining showed a punctate pattern of p62 and the accumulation of aggresomes (Appendix Fig S5C). These autophagy defects could be related to mTOR pathway hyperactivation (Appendix Fig S5D and E).

Our findings suggest that defective autophagy and consequent p62 accumulation are common features of loss-of-function mutations of the three known CCM genes.

### Enhanced p62 accumulation occurs in endothelial cells lining in human CCM lesions

To examine the clinical relevance of our findings in cellular and animal models of CCM disease, we analyzed p62 expression in human CCM lesions. Indeed, p62 accumulates in several autophagy-deficient mouse tissues (Zatloukal *et al*, 2002; Martinet *et al*, 2013) and represents a reliable marker for tissues with reduced autophagic activity (Waguri & Komatsu, 2009).

**Figure 4:**
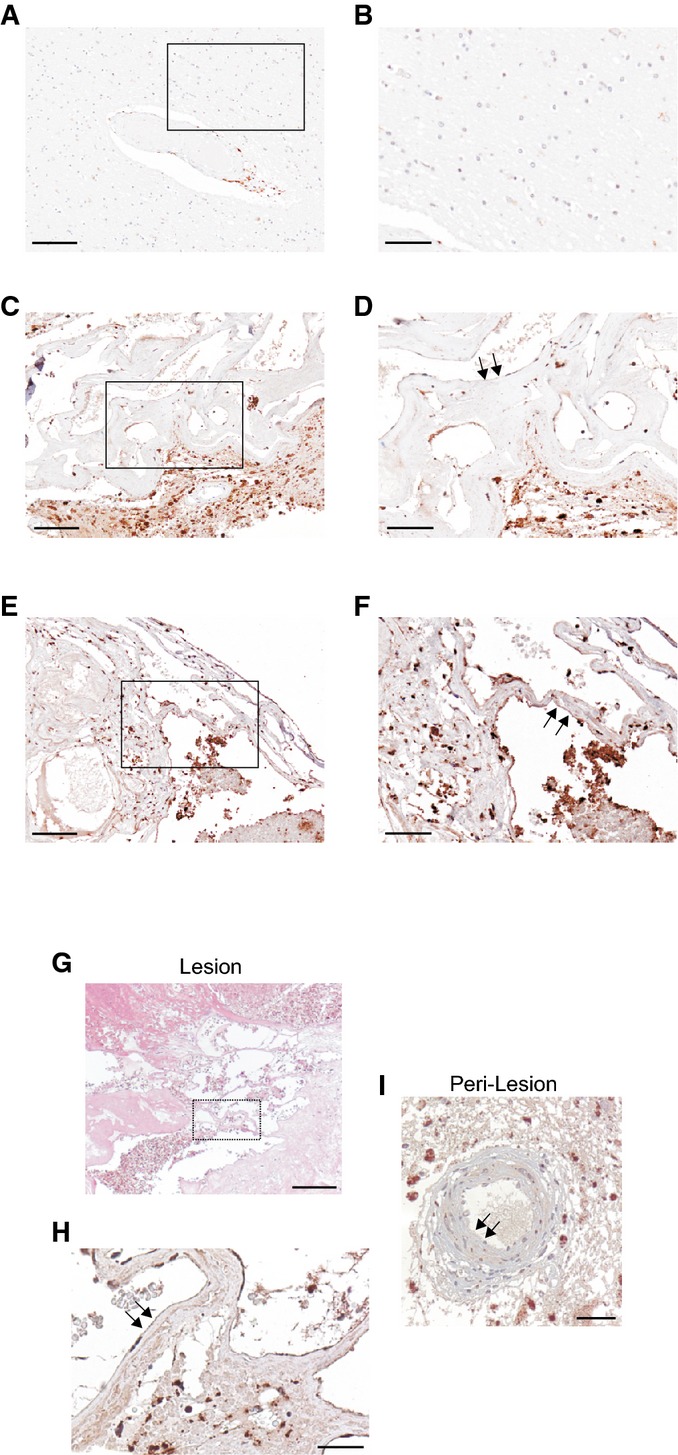
Accumulation of p62 in endothelial cells lining human CCM lesions p62 immunohistochemical (IHC) staining in human brain tissue. A, B Normal vascular endothelium of autoptic brain parenchyma samples is lacking the typical autophagic p62 granules as shown by the negative staining for p62. Scale bars: (A) 200 μm; (B) 100 μm.

Histological samples of human CCM lesions were obtained from archived paraffin-embedded surgically resected CCM specimens, and p62 levels were evaluated by immunohistochemical studies. The analysis of CCM specimens from 10 cases with confirmed diagnosis of CCM by both neuroradiological and histopathological analyses revealed enhanced staining intensity for p62 in endothelial cells lining CCM lesions (Appendix Table S1). Representative immunohistochemical results for the selected cases are shown in Fig4. While normal brain vascular endothelium deriving from autoptic samples showed negative staining for p62 (Fig4A and B), either moderate (Fig4C and D) or marked (Fig4E and F) “pearl necklace-like” endothelial staining for p62 was observed in the ten CCM cases analyzed (Fig4C–F and Appendix Table S1). Intriguingly, a putative association between marked p62 accumulation and the multiple CCM lesion phenotype was also observed (Appendix Table S1), which deserves further investigation in larger samples for validation. Notably, in one of the eight tissue samples that displayed marked positive p62 staining in CCM lesions, typical normal vessels surrounding the lesion were also present and stained negative for p62, resulting in an internal negative control (Fig4G–I).

C–F Two different representative samples of CCM lesions with a thin, single layer brain endothelium displaying either moderate (C, D) or marked (E, F) positive perinuclear “pearl necklace-like” immunostaining for p62 granules. (C, D), case n° 4 (p62^++^), and (E, F), case n° 8 (p62^+++^) are representative of CCM cases listed in Appendix Table S1. Scale bars: (C, E) 200 μm; (D, F) 100 μm. Arrows indicate endothelial p62 positive staining.

G–I Hematoxylin and eosin (H&E) staining (G) and p62 immunohistochemical analysis (H, I) of a CCM surgical sample (case n° 6 in Appendix Table S1) containing normal vessels in the peri-lesional area, which served as an internal control. Notice marked p62-positive staining in endothelial cells lining a CCM lesion (H, arrows) and p62-negative staining in endothelial cells lining a normal peri-lesional vessel (I, arrows). Scale bars: (G) 300 μm; (H, I) 100 μm. Background staining in brain parenchyma surrounding CCM lesions may be attributed to either cell debris or p62 immunoreactivity in neuronal and glial cells.

Taken together, these data demonstrate that p62 accumulates in endothelial cells lining CCM brain lesions, supporting the clinical relevance of defective autophagy in CCM disease.

In conclusion, we identified a key role for autophagy inhibition in CCM pathogenesis and suggest the utilization of mTOR inhibitors, which are currently used in several clinical trials, including the treatment of complicated vascular anomalies (Lackner *et al*, 2015), as a promising therapeutic approach for treating CCM disease. Recent observations regarding the role of mTOR in arteriovenous malformations (Kawasaki *et al*, 2014), the higher number and tortuosity of tumor microvessels in Atg5^EC-KO^ mice carrying an endothelial cell-specific deletion of the autophagy-related gene Atg5 (Maes *et al*, 2014), and the involvement of autophagy in CCM3-dependent senescence induction (Guerrero *et al*, 2015) provide further support to our data, strengthening the original finding that CCM is an autophagy-related disease.

## Materials and Methods

### Immunoblotting

For immunoblotting, cells were scraped into ice-cold, phosphate-buffered saline (PBS) and lysed in a buffer containing 50 mM Tris HCl pH 7.4, 150 mM NaCl, 1% Triton X-100, 0.2% SDS, protease, and phosphates inhibitor cocktail. After 30 min of incubation on ice and centrifugation at 2,500 rpm 4°C for 5 min, proteins were quantified by the Lowry method and 10 μg of each sample were loaded on a Novex NuPage Bis-Tris 4–12% precast gel (Life Technologies) and transferred to PVDF membranes. After incubation with TBS–Tween-20 (0.05%) supplemented with 5% non-fat powdered milk for 1 h to saturate unspecific binding sites, membranes were incubated overnight with primary antibodies. The revelation was assessed by appropriate horseradish peroxidase-labeled secondary antibodies (Santa Cruz Biotechnology), followed by detection by chemiluminescence (ThermoScientific), using ImageQuant LAS 4000 (GE Healthcare).

### Antibodies

For immunofluorescence and Western blotting, the following primary antibodies were used: rabbit anti-p62 [P0067] (1:2,000 for Western blot; 1:100 for immunofluorescence), mouse anti-β-actin [A1978] (1:10,000), rabbit anti-LC3B [L7543] (1:1,000), rabbit anti-CCM2 [HPA020273] (1:1,000), and rabbit anti-AMBRA1 [PRS4555] (1:1,000) from Sigma-Aldrich; rabbit anti-GAPDH [#2118] (1:5,000), rabbit anti-mTOR [#2983] (1:1,000), rabbit anti-phospho-mTOR (Ser 2448) [#5536] (1:1,000), rabbit anti-p70 S6 Kinase [#9202] (1:1,000), rabbit anti-phospho-p70 S6 Kinase (Ser 371) [#9208] (1:1,000), rabbit anti-4E-BP1 [#9644] (1:1,000), rabbit anti-phospho-4E-BP1 (Thr 37/46) [#2855] (1:1,000), rabbit anti-ULK1 [#8054] (1:1,000), and rabbit anti-phospho-ULK1 (Ser 757) [#6888] (1:500) from Cell Signaling; rabbit anti-phospho-AMBRA1 (Ser 52) [#ABC80] (1:1,000) from Millipore; rabbit anti-alpha-SMA [NB 600-531] (1:1,000) from NovusBio; mouse anti-N-cadherin [33–3900] (1:500) from Invitrogen; goat anti-CD31/Pecam-1 [sc-1506] (1:1,000), mouse anti-LAMIN A/C [sc-7292] (1:1,000), and mouse anti-VE-cadherin [sc-9989] (1:500) from Santa Cruz Biotechnology; and rabbit anti-KRIT1 (1:500) from S.F. Retta.

### Reagents

Chemicals used were the following: N-acetylcysteine (NAC; Sigma-Aldrich), Bafilomycin A1 (BafA1; Sigma-Aldrich), Torin1 (Torin1; Calbiochem), Rapamycin (Rapa; Calbiochem), and Cycloheximide (CHX; Sigma-Aldrich).

### Cell cultures and transfections

KRIT1 wt, KRIT1 KO, and KRIT1 KO re-expressing KRIT1 mouse embryonic fibroblasts (MEFs) were provided by S.F. Retta (Goitre *et al*, 2010) and cultured in a humidified 5% CO_2_, 37°C incubator in Dulbecco’s modified Eagle’s medium (DMEM) supplemented with 10% fetal bovine serum (FBS; Life Technologies), 2 mM l-glutamine, 100 U/ml penicillin (EuroClone), and 100 mg/ml streptomycin (EuroClone).

KRIT1 wt, KRIT1 KO, CCM3 wt, and CCM3 KO endothelial cells were provided by E. Dejana. Endothelial cells were cultured in a humidified 5% CO_2_, 37°C incubator on 0.1% gelatin-coated 75 cm^2^ Falcon flasks in MCDB 131 Medium (Life Technologies) supplemented with 20% FBS, 2 mM l-glutamine, 1 mM sodium pyruvate, 100 U/ml penicillin and 100 mg/ml streptomycin, 100 μg/ml heparin, and 50 μg/ml Endothelial Cell Growth Supplement (ECGS, Sigma-Aldrich). Transient transfections were performed using JetPEI (Polyplus transfection™) and Lipofectamine 2000 (Life Technologies) as transfecting reagents, according to the manufacturer’s instructions.

Human umbilical vein endothelial cells (HUVECs) were purchased from Life Technologies and cultured in Medium 200 supplemented with low serum growth supplement (LSGS). The cells in the present study were used in passages 2–6. Transfections were performed using Lipofectamine RNAiMax (Life Technologies) as transfecting reagent, according to the manufacturer’s instructions. ATG7 and negative siRNAs were purchased from Cell Signaling.

The human cerebral microvascular endothelial cells (hBMEC) were purchased from ScienceCell Research Laboratory (Carlsbad). The hBMECs were grown in EGM-2MV medium (Lonza). Cells were grown on 6-well plates and coated with rat tail collagen type-I (BD Biosciences). The human umbilical vein cell line, EA.hy926, established by fusing primary human umbilical vein cells with a thioguanine-resistant clone of A549 was purchased by ATCC and cultured in Dulbecco’s modified Eagle’s medium (DMEM—Sigma-Aldrich, St Louis, MO, USA) supplemented with 10% FBS, 2 mM L-glutamine, and 1% penicillin/streptomycin. The cells were maintained in a 37°C incubator in a humidified atmosphere containing 5% CO_2_.

hBMEC cells (2.5 × 10^5^ per well) were subjected to two round of transfection with siRNA targeting KRIT1 or a scrambled control. Briefly, cells were passaged into 25 nM siRNA with 1:166 HiPerFect reagent (Qiagen) in 4:1 EGM-2MV, respectively, and plated. After an overnight incubation in the transfection mix, cells were washed and fed with EGM-2MV. After an additional 48 h, the transfection process was repeated to achieve more complete knockdown. During the second transfection, cells were seeded into assay plates as described below. Cells were again fed with EGM-2MV completed medium after overnight incubation with the transfection mix. After an additional 48–72 h, cells were subjected to experimental conditions.

EA.hy926 endothelial cells were plated in 10-cm culture dishes in 8 ml antibiotic-free standard growth medium supplemented with FBS. Cells were grown to 60% confluence and then transfected for 5 h at 37°C with KRIT1 or control siRNAs (final concentration: 100 nmol/l). Specifically, silencing experiments were performed using a mix of 4x pre-designed iBONi siRNA against KRIT1 target gene. IBONi positive and negative controls were purchased from Ribbox Life Sciences. Cell transfections were performed using INTERFERin kit (Polyplus transfection) according to the manufacturer’s protocol. Cells were cultured with siRNAs for 24 h before treatments and analysis.

For *CCM2*-silencing experiments, transfections were performed using Lipofectamine RNAiMax (Life Technologies) as transfecting reagents, according to the manufacturer’s instructions. CCM2 and negative siRNAs (final concentration 40 nM) were purchased from Life Technologies.

### Immunohistochemical analysis

The study was performed according to the standards of the Institutional Ethical Committee and the Helsinki Declaration and was approved by the Institutional Review Board of our hospital. Specifically, approval was given by the ethic institutional review board for “Biobanking and use of human tissue for experimental studies” of the Pathology Services (Azienda Ospedaliera Citta della Salute e della Scienza di Torino and Department of Medical Sciences of the University of Torino). All patient records were anonymized and de-identified prior to analysis.

Histological samples of human CCM lesions were obtained from archived, formalin-fixed, paraffin-embedded surgically resected CCM specimens retrieved from the Department of Anatomy and Diagnostic Histopathology at the “Città della Salute e della Scienza” University Hospital, Turin, Italy. At the time of neurosurgery, an informed consent was asked by neurosurgeons to patients (or legal representatives) for scientific use of residual materials according to Institutional Rules defined by the Ethical Committee of the “Città della Salute e della Scienza” University Hospital, Turin. Only archived specimens with confirmed diagnosis of CCM by both neuroradiological and histopathological analyses were included in the study. Most of the selected CCM specimens were linked to patients carrying multiple CCM lesions, which are considered a marker of hereditary CCM, suggesting that they could represent the familial form of the disease. However, whereas some specimens derived from carriers of a single CCM lesion, which might be suggestive of a sporadic case, neither documented family history information nor genetic data were available from most of the corresponding medical records.

Histological serial sections (3 μm thick) of selected paraffin-embedded CCM specimens were prepared and routinely stained with hematoxylin and eosin (H&E). Two pathologists (A.P. and P.C.) independently reviewed the H&E-stained slides, whereas additional sections, collected on superfrost plus slides, were used for immunohistochemical analysis.

Immunohistochemical reactions were performed referring to the following protocol: briefly, histological sections were deparraffinized, rehydrated, and subjected to a 30-min cycle at boiling temperature in citrate buffer (pH 6.0) for antigen retrieval. Endogenous peroxidase activity was blocked by a 7-min incubation with H_2_O_2_ solution RPE 6%. Thereafter, the sections were incubated for 45 min with guinea pig monoclonal anti-p62 primary antibody (diluted 1:300, Progen Biotechnik), followed by incubation with an HRP-labeled polymer conjugated secondary antibody (1:800, Santa Cruz Biotechnology) for 30 min at RT. Labeling was then visualized by a 5- to 10-min incubation with 3,3′ diaminobenzidine + H_2_O_2_ substrate chromogen which results in a brown-colored precipitate at antigen site. The sections were subsequently counterstained with hematoxylin. Immunohistochemical variables were scored by evaluating the percentage of stained perinuclear regions at a 40× magnification in endothelial lumens.

### Immunofluorescence

Cells, transfected or treated as described, were washed with PBS and fixed with 4% formaldehyde for 10 min at room temperature. After washing three times with phosphate-buffered saline (PBS), cells were permeabilized with 0.1% Triton X-100 in PBS (PBST) at room temperature for 10 min and blocked with PBST containing 5% BSA at room temperature for 1 h. Cells were incubated with primary antibody in PBST containing 5% BSA overnight at 4°C, washed three times with PBS, and then incubated with appropriate isotype-matched, AlexaFluor-conjugated secondary antibodies (Life Technologies) at room temperature for 1 h. Digital images were acquired with confocal microscope (Zeiss LSM510) using a 63 × 1.4 NA Plan-Apochromat oil-immersion objective. Acquired images were then analyzed by using open source software Fiji.

### Immunostaining for fluorescence microscopy of brain sections and retinas

All animal procedures were performed in accordance with the Institutional Animal Care and Use Committee (IACUC), in compliance with the guidelines established in the Principles of Laboratory Animal Care (directive 86/609/EEC) and approved by the Italian Ministry of Health.

Brains and eyes from mice pups were fixed in 3% paraformaldehyde immediately after dissection, and fixation was continued overnight at 4°C. The retinas were dissected from the eyes just before staining as the whole mount. Fixed brains were embedded in 4% low-melting-point agarose and sectioned along the sagittal axis (150 μm) using a vibratome (1000 Plus, The Vibratome Company, St. Louis, MO, USA).

Brain sections and retinas (as whole mount) were stained as floating samples in 12-well and 96-well plates, respectively. They were blocked overnight at 4°C in 1% fish-skin gelatin with 0.5% Triton X-100 and 5% donkey serum in phosphate-buffered saline (PBS) containing 0.01% thimerosal. The samples were incubated overnight at 4°C with the primary antibodies diluted in 1% fish-skin gelatin with 0.25% Triton X-100 in PBS containing 0.01% thimerosal. Following washing with 0.1% Triton X-100 in PBS, the secondary antibodies were added for 4 h at room temperature in 1% fish-skin gelatin with 0.25% Triton X-100 in PBS containing 0.01% thimerosal. The incubation with DAPI was in PBS for 4 h, which was followed by several washes in PBS, post-fixation with 3% paraformaldehyde for 5 min at room temperature, and further washes in PBS. The brain sections were mounted in Vectashield with DAPI, and the coverslips fixed with nail varnish; the retinas were mounted in Prolong gold with DAPI.

### CCM3-ECKO mice

*CCM3-flox/flox* mice were bred with *Cdh5(PAC)-CreERT2* mice for Tamoxifen-inducible endothelial cell-specific expression of Cre recombinase and *CCM3* gene recombination.

*CCM3-flox/flox* mice: these mice were generated at TaconicArtemis (Koeln, Germany) on a C57BL/6N background according to the knock-in procedures. In this case, two P-lox sequences were inserted that flank exons 4 and 5 of the murine *CCM3* gene. P-lox sites can be targeted by the Cre recombinase enzyme, which induces recombination and subsequent excision of the nucleotides inserted between P-lox sequences. These mice have been used to control in a time-dependent manner for the deletion of *CCM3* gene.

*Cdh5(PAC)-CreERT2* mice175: these mice have been kindly donated by Dr. R.H. Adams, (University of Munster, Munster, Germany) and present the CreERT2 gene under the VE-cadherin (Cdh5) promoter. Since VE-cadherin is an endothelial-specific gene, the expression of CreERT2 is confined to the endothelial district. CreERT2 gene expresses a fusion protein in which Cre recombinase has been fused together with the regulatory domain of the estrogen receptor. This domain is able to retain Cre recombinase into the cytoplasm of ECs in resting conditions. Only after stimulation with estrogen or an analog such as Tamoxifen CreERT2, fusion protein can dimerize and translocate to the nucleus where it can act on P-lox sites to drive homologous recombination. This model allows the operator to induce Cre recombinase expression in an endothelial-specific fashion just by Tamoxifen administration.

### mRFP-GFP-LC3 detection

For autophagic flux measurements, cells cultured on 24-mm glass coverslips were transfected with the mRFP-GFP-LC3 tandem construct. After 24 h of expression, cells were placed in an open Leyden chamber on a 37°C thermostated stage. The quantitative analysis of the autophagic flux was performed on a Nikon LiveScan Swept Field Confocal Microscope (*SFC*) Eclipse *Ti* with a 60× magnification and equipped with NIS-Elements microscope imaging software (Nikon Instruments). For each condition, the colocalization of the red and green signals was determined by manual counting of fluorescent puncta in at least 20 independent visual fields.

### ROS measurements using mt-HyPer probe

For mitochondrial H_2_O_2_ levels measurements, KRIT1 KO and KRIT1 KO re-expressing KRIT1 cells were cultured on 24 mm glass coverslips and transfected with the H_2_O_2_ sensor pHyPer-dMito (mt-HyPer). After 24 h of expression, cells were maintained in Krebs–Ringer buffer (KRB: 135 mM NaCl, 5 mM KCl, 1 mM MgSO_4_, 0.4 mM KH_2_PO_4_, 5.5 mM glucose, 20 mM HEPES, pH 7.4), supplemented with 1 mM CaCl_2_, and placed in an open Leyden chamber on a 37°C thermostated stage. 494/406 nm excitation filters and a 500-nm long-pass beam splitter were used, and an image pair was obtained in every 200 ms. The fluorescence data collected were expressed as emission ratios. The experiments were performed on Cell^R multiple wavelength high-resolution fluorescence microscopy system.

### Soluble/Insoluble fraction

Total cellular proteins were separated into detergent-soluble and detergent-insoluble fractions. For detergent-soluble fraction, cells were lysed with a 1% Triton X-100 buffer (50 mM Tris pH 8.0, 150 mM NaCl, 1 mM EDTA, 10% glycerol, 1% Triton X-100, protease, and phosphates inhibitor cocktail). After 30 min on ice, lysates were centrifuged for 15 min at 12,000 *g* at 4°C and supernatant was collected as detergent-soluble fraction. The pellet was solubilized in the same buffer with addition of 1% SDS for 10 min at RT and, after centrifugation at 12,000 *g* for 15 min, the supernatant was collected as detergent-insoluble fraction. The detergent-soluble (10 μg) and detergent-insoluble (5 μg) fractions were subjected to immunoblot analysis. GAPDH and Lamin A/C were used as controls of the detergent-soluble and detergent-insoluble fractions, respectively.

### Real-time PCR

Total RNA was extracted with TRIzol® Reagent (Invitrogen, Carlsbad, CA, USA). RNAs were purified with RNeasy Mini Kit (Qiagen GmbH, Hilden, Germany), and DNase digestion was performed with RNase-Free DNase Set (Qiagen). The RNA quality and concentration were measured using the NanoDrop™ ND-1000 (Thermo Scientific).

For the first-strand cDNA synthesis, 1,000 ng of total RNA of each sample was reverse-transcribed with M-MLV Reverse Transcriptase (Invitrogen), following the manufacturer’s protocol.

Human primers were selected for each target gene with Primer 3 software. Real-time PCRs were carried out using the designed primers at a concentration of 300 nM and FastStart SYBR Green Master (Roche Diagnostics, Mannheim, Germany) on a Rotor-Gene 3000 (Corbett Research, Sydney, Australia). Thermal cycling conditions were as follows: 10-min denaturation at 95°C, followed by 40 cycles of denaturation for 10 s at 95°C; annealing for 20 s at 60°C; and elongation for 30 s at 72°C. Values were normalized to the expression of the glyceraldehyde-3-phosphate dehydrogenase (GAPDH) and glucuronidase beta (GUSB) internal references, whose abundance did not change under our experimental conditions.

### Cell proliferation assay

Cell proliferation assays were performed using a colorimetric method based on crystal violet staining. Cells, seeded at a density of *n* cells per well in 12-well plates, are left untreated or treated with Rapamycin (500 nM) or Torin1 (100 nM) in complete medium at 37°C for different time periods. Starting from the following day (day 1), 1 set of wells (at days 2, 3, 4) was washed with PBS, fixed for 10 min at room temperature in 4% formaldehyde, and then left in PBS at 4°C. The last day, all the wells were washed with PBS and then stained with crystal violet for 20 min. Excess dye was removed by three washes with PBS, and plates were allowed to air dry. After solubilization with 10% acetic acid solution, the absorbance was read at 595 nm with a microplate reader (SPECTROstar Nano-BMG Labtech).

### Aggresome detection

The ProteoStat Aggresome Detection Kit (Enzo Life Sciences) was used according to the manufacturer’s instructions. The kit provides a 488-nm excitable red fluorescent molecular rotor dye for the specific detection of aggregated proteins and aggresome-like inclusion bodies in fixed and permeabilized samples.

### Tube formation assay

HUVECs (2 × 10^5^ cells) transfected with scrambled siRNA or *ATG7* siRNA were seeded onto 6-cm culture dishes coated with BD Matrigel™ Matrix (Becton-Dickinson) and incubated for 6 h at 37°C. After that, the formation of capillary-like structures was stained with calcein (Life Technologies) for 20 min at 37°C and then observed using an Axiovert 200M Carl Zeiss fluorescence microscope (20× objective); phase-contrast images were scanned with Leica DM IL LED, using a 4× objective.

### Endothelial cell migration assay

Endothelial cell migration was detected by Transwell chamber assay (Corning). Briefly, the medium containing a 5% FBS was added to the lower chambers, and then, cells (HUVEC or lung endothelial cells) were suspended in 100 μl of serum-free medium and seeded onto the upper chambers. After incubation at 37°C for 8 h, the migrated cells were fixed with 4% paraformaldehyde, stained with the nuclear marker DAPI, and then photographed and automatically counted using a custom-made Cell Profiler pipeline. The images have been taken with a Carl Zeiss Axiovert 200 M using a 20× magnification.

### Statistical analysis

Statistical analyses were performed using an unpaired two-tailed *t*-test (two groups) or one-way ANOVA with Bonferroni correction (for groups of three or more). For grouped analyses, multiple unpaired *t*-test with correction for multiple comparisons using the Holm–Sidak method was performed. Normal distribution of data was assessed by applying a D’Agostino & Pearson omnibus normality test. *F*-test was used to compare variances between groups. A *P*-value < 0.05 was considered significant. All data are reported as mean ± s.e.m. Exact *P*-values are indicated in the figure legends.

**The paper explained**

**Problem**

Cerebral Cavernous Malformations (CCM, also known as cavernous angioma or cavernoma) are major vascular malformations having a raspberry-like appearance and consisting of closely clustered, abnormally dilated and leaky capillary channels (caverns). Within the brain, CCMs occur as single or multiple lesions and, depending on the size and location, can be clinically silent or give rise to serious clinical symptoms such as headaches, neurological deficits, seizures, stroke, and intracerebral hemorrhage that can result in death. CCM is a disease of proven genetic origin that may arise sporadically or can be inherited as an autosomal dominant condition with incomplete penetrance and variable expressivity. Genetic studies have so far identified three genes whose mutation causes CCM: KRIT1 (CCM1), CCM2 and CCM3. To date, there are not direct therapeutic approaches for the CCM disease, besides the surgical removal of accessible lesions in patients with recurrent hemorrhage or intractable seizures. In particular, novel pharmacological strategies are required for preventing the *de novo* formation of CCM lesions in susceptible individuals and the progression of the disease. A deep understanding of the molecular mechanisms underlying CCM disease pathogenesis should provide a fundamental framework for the development of novel, more safe therapeutic strategies, specially required for inoperable or multiple lesions.

**Results**

Malfunction of autophagy, one of the major degradative processes inside the cell, is often related to a plethora of human diseases. We found that down-regulation of CCM genes suppressed autophagy both in cellular and animal models of CCM disease. Moreover, we show that human CCM lesions display increased levels of p62/SQSTM1, an autophagic marker that accumulates when autophagy is inhibited. Furthermore, suppression of autophagy is linked to the upregulation of the mTOR regulatory pathway. In addition, we demonstrated that defective autophagy is related to endothelial-to-mesenchymal transition and ROS production, two key phenotypic signatures of CCM disease. Notably, pharmacological inhibition of mTOR significantly increased autophagy and rescued some of the molecular and cellular phenotypes associated with CCM.

**Impact**

Through the identification of defective autophagy as a potential key aspect in the pathogenesis of CCM disease, we offer initial clues and novel options for the development of therapeutic strategies based on autophagy modulation. In particular, our findings suggest that targeting the mTOR pathway may be a reasonable strategy to alleviate clinical symptoms caused by CCM lesions. Indeed, mTOR inhibitors (such as Rapamycin) are currently used in several clinical trials, including treatment of vascular anomalies, and are usually well tolerated. Thus, they might represent novel therapeutic agents in the treatment of CCM disease.

## For more information

- www.unife.it/labs/signaltransduction: Paolo Pinton’s Lab website
- http://www.ccmitalia.unito.it/: CCM Italia, the Italian multidisciplinary research network on Cerebral Cavernous Malformation (CCM) disease.
- http://www.ccmitalia.unito.it/aiac/: Associazione Italiana Angiomi Cavernosi (AIAC), the Italian patient association on Cerebral Cavernous Malformation (CCM) disease committed to providing science-based and community-driven information and support for Italian people affected by cavernous angiomas.
- http://www.angiomaalliance.org/: Angioma Alliance, an organization by and for those affected by cavernous angiomas and their loved ones, health professionals, and researchers.
- http://www.cavernoma.org.uk: Cavernoma Alliance UK, a charity created to improve the quality of life for those affected by cavernous angiomas through education, support, and promotion of research.
- https://www.telethon.it/ricerca-progetti/malattie-trattate/malformazione-cavernosa-cerebrale.
- http://www.omim.org/entry/116860: Cerebral Cavernous Malformation (CCM) disease, a Mendelian vascular disorder (OMIM 116860) with an estimated prevalence of 0.3–0.5% in the general population.

## Supporting Information

**Appendix**

**Review Process File**
