# Supplementary material for: Defective autophagy is a key feature of cerebral cavernous malformations
Source: EMBO Mol Med. 2015 Sep 28;7(11):1403–17. doi: 10.15252/emmm.201505316 (PMC4644374; doi:10.15252/emmm.201505316)
Supplement: Supplementary file 1 [file emmm0007-1403-sd1.pdf]

Appendix

Table of Contents:

- Appendix Table S1
- Appendix Figure S1
- Appendix Figure S2
- Appendix Figure S3
- Appendix Figure S4
- Appendix Figure S5
- Appendix Figure Legends

Cases	Histological number	Stain Intensity	Multiple Lesions	Single large Lesion
1	2554/2013	+++	X	
2	8189/2012	+++	X	
3	6292/2003	+++	X	
4	9008/2012	++		X
5	2414/2007	+++	X	
6	7369/2007	++		X
7	760/2004	+++	X	
8	9368/2010	+++	X	
9	6577/2011	+++	X	
10	10490/2012	+++	X	

Appendix Table S1

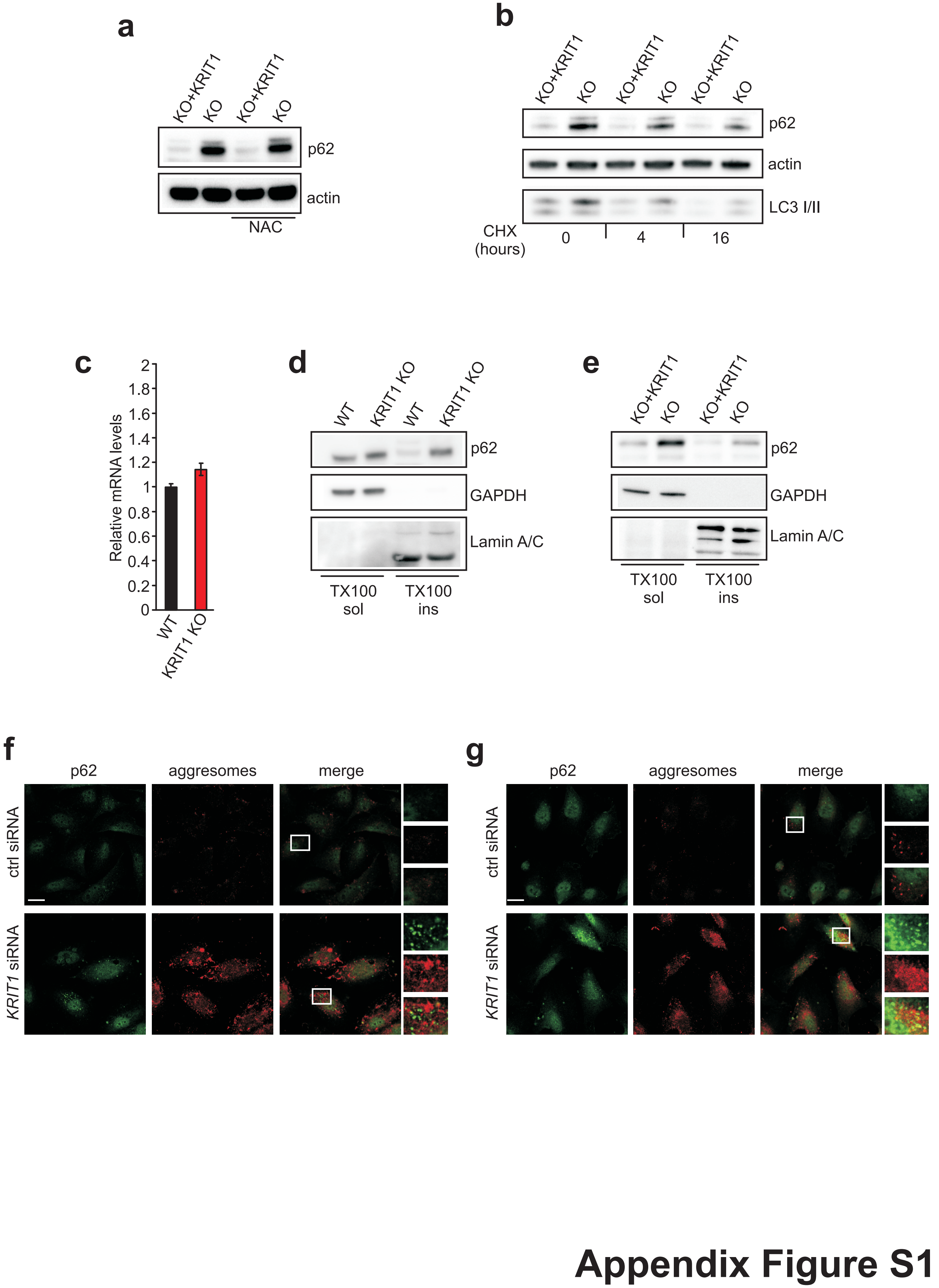

Appendix Figure S1

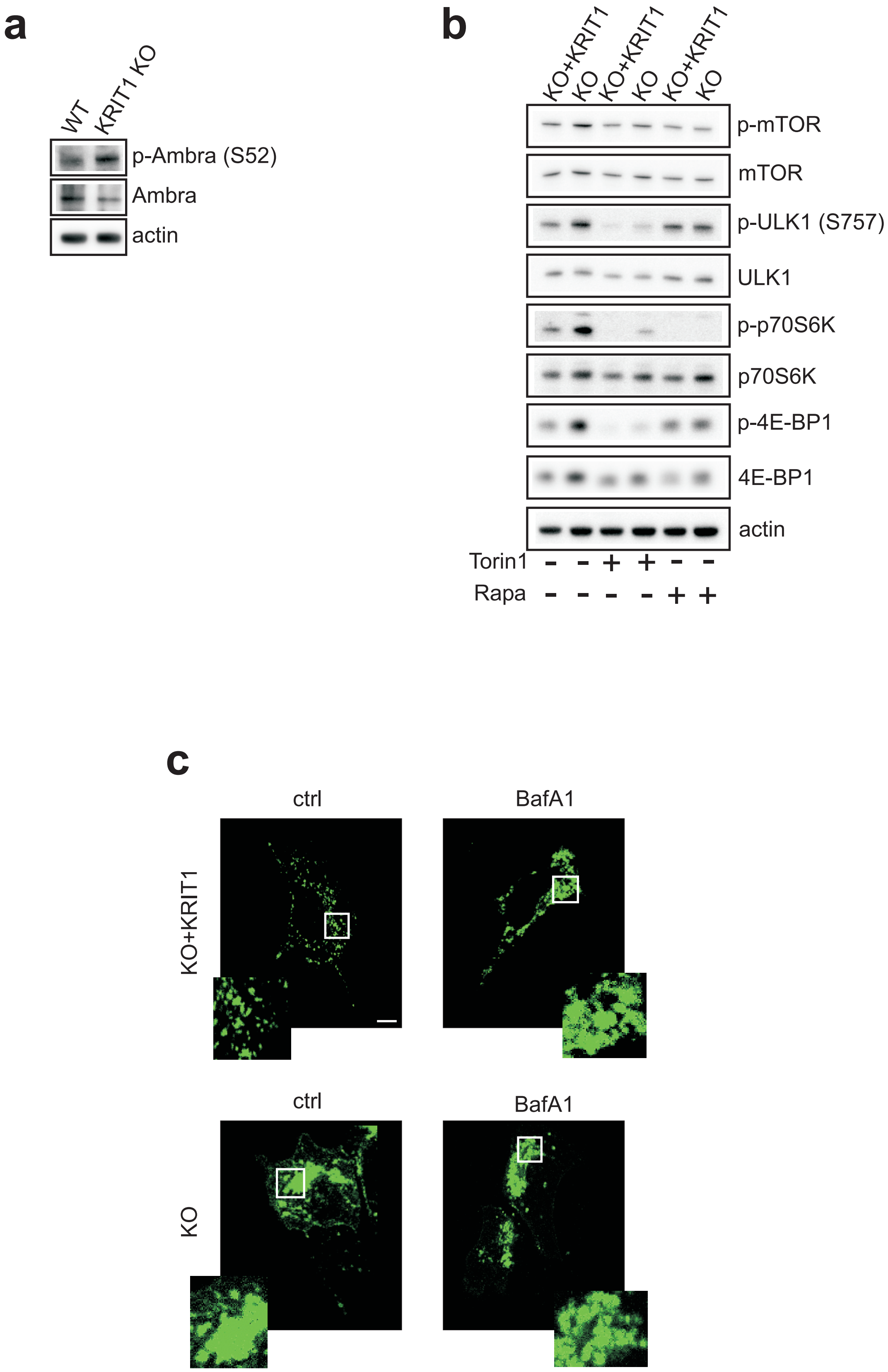

Appendix Figure S2

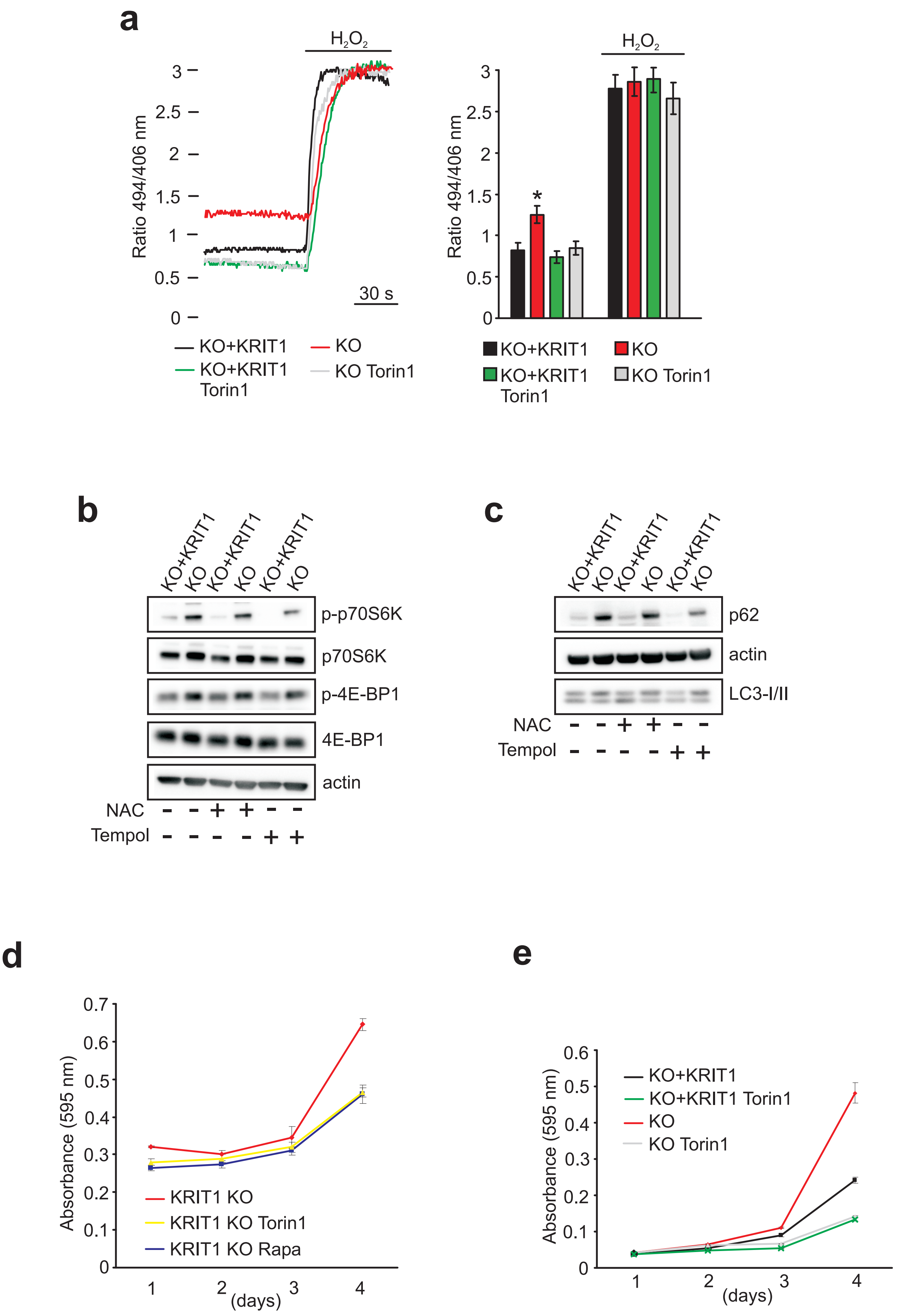

Appendix Figure S3

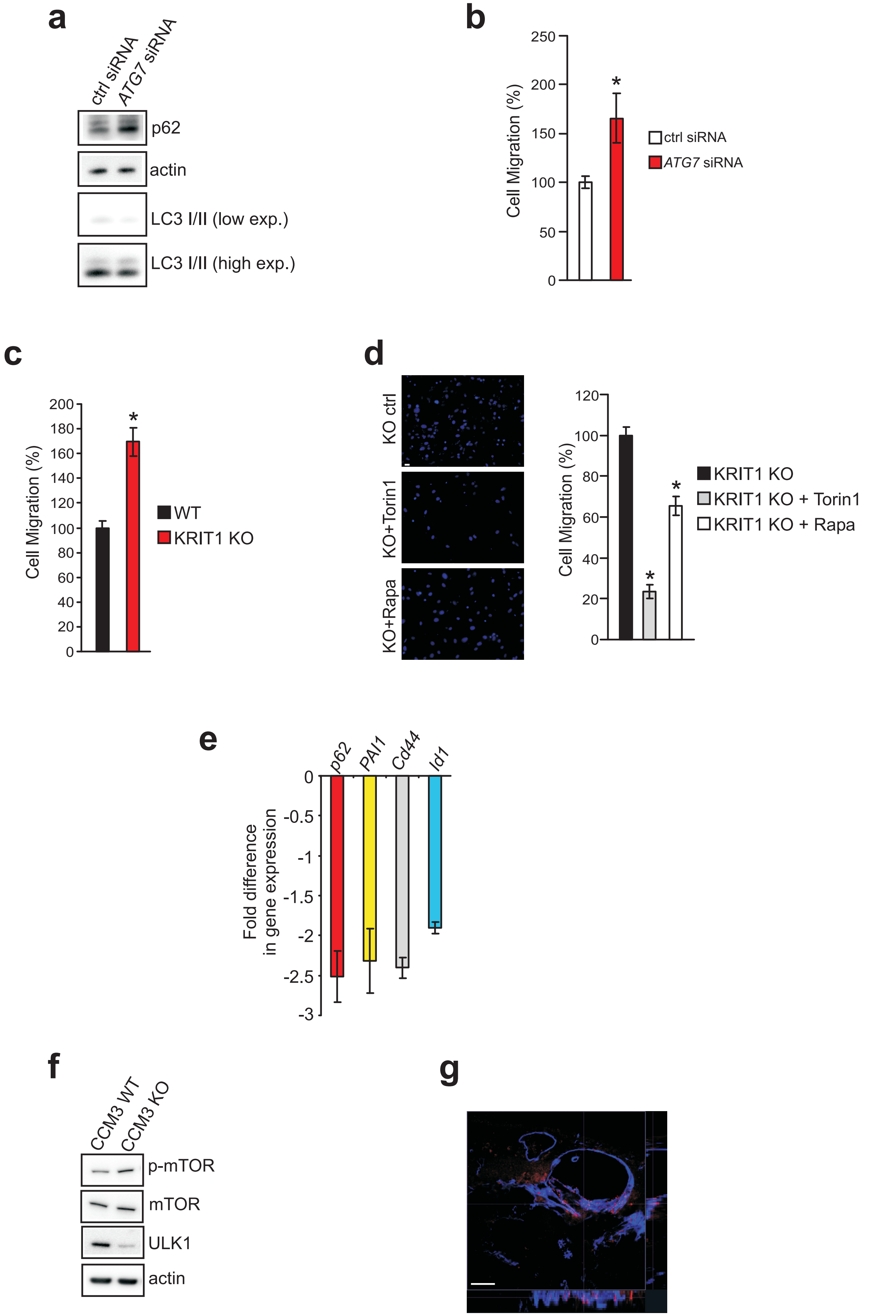

Appendix Figure S4

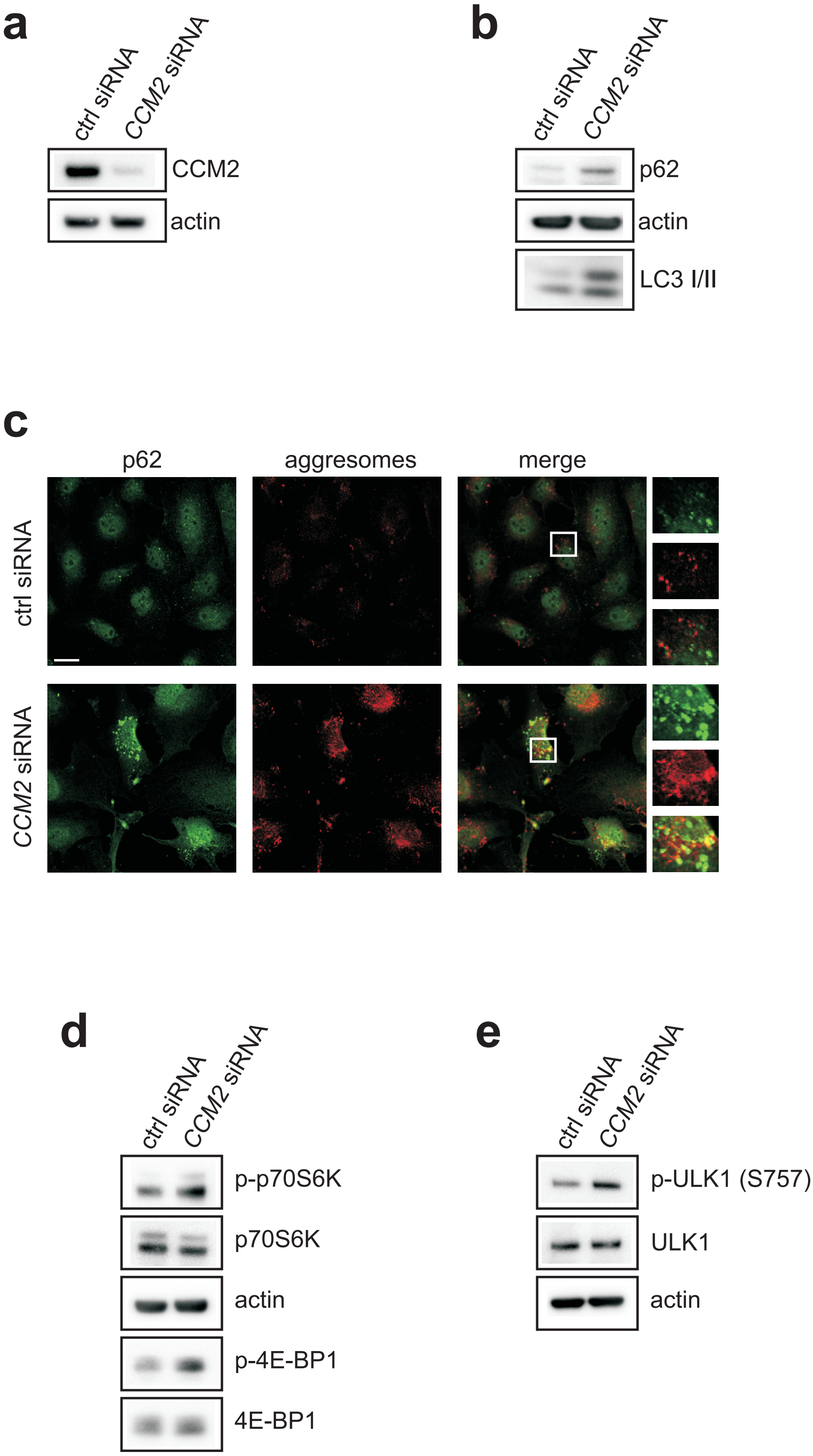

Appendix Figure S5

Appendix Table S1:

Clinical records related to CCM specimens used in this study.

Formalin-fixed, paraffin-embedded surgically resected CCM specimens were retrieved from the archives of the Department of Anatomy and Diagnostic Histopathology at the "Città della Salute e della Scienza" University Hospital, Turin, Italy. Only archived specimens with confirmed diagnosis of CCM by both neuroradiological and histopathological analyses were included in the study.

Appendix Figure S1:

(**a**) Immunoblot analysis with specific antibody to p62 and actin. Where indicated, KO+KRIT1 and KRIT1 KO MEFs were treated with 20mM NAC for 24 hours. Results are representative of three independent experiments.

(**b**) Immunoblot analysis of p62 and LC3-I/II in CCM1 KO re-expressing KRIT1 (KO+KRIT1) and KRIT1 KO MEFs, treated or not with cycloheximide (CHX) for 4 or 16 hours. Actin was used as loading marker. Results are representative of three independent experiments.

(**c**) Gene expression levels of *p62* in WT and KRIT1 KO endothelial cells. Data are mean ± s.e.m of three independent experiments.

(**d**) Immunoblot analysis of p62 in detergent-soluble (TX-100 sol) and -insoluble (TX-100 ins) fractions from WT and KRIT1 KO endothelial cells. GAPDH and LAMIN A/C were used as loading markers for soluble and insoluble fractions, respectively. Results are representative of three independent experiments.

(**e**) Immunoblot analysis of p62 in detergent-soluble (TX-100 sol) and -insoluble (TX-100 ins) fractions from KO+KRIT1 and KRIT1 KO MEFs. GAPDH and LAMIN A/C were used as loading markers for soluble and insoluble fractions, respectively. Results are representative of three independent experiments.

(**f**) Immunofluorescence analysis of p62 (green) and ProteoStat Aggresome staining detection reagent (red) in hBMEC cells, transfected with negative siRNA (ctrl siRNA) or *KRIT1* siRNA. The yellow signal in the merged images represents an overlapping spatial relationship between green and red fluorescence. Magnification in insets. Scale bar, 20 μm. The images are representative of three independent experiments.

(**g**) Immunofluorescence analysis of p62 (green) and ProteoStat Aggresome staining detection reagent (red) in EA.hy926 cells, transfected with negative siRNA (ctrl siRNA) or *KRIT1* siRNA.

The yellow signal in the merged images represents an overlapping spatial relationship between green and red fluorescence. Magnification in insets. Scale bar, 20 μm. The images are representative of three independent experiments.

Appendix Figure S2*:*

(**a**) Immunoblot analysis of phospho-AMBRA1 (Serine 52), AMBRA1 and actin in WT and KRIT1 KO endothelial cells. Results are representative of three independent experiments.

(**b**) Immunoblot analysis of phosphorylated mTOR (Ser 2448), total mTOR, phosphorylated p70 S6 Kinase (Ser 371), total p70 S6 Kinase, phosphorylated 4E-BP1 (Thr 37/46), total 4E-BP1 and actin (loading marker). KRIT1 KO re-expressing KRIT1 (KO+KRIT1) and KRIT1 KO cells were treated or not with 100nM Torin1 or 500nM Rapamycin for 4 hours. Results are representative of three independent experiments.

(**c**) Confocal microscopy of KRIT1 KO re-expressing KRIT1 (KO+KRIT1) and KRIT1 KO MEFs, transiently transfected with the lysosomal marker LAMP1-GFP. Where indicated, cells were treated with 1nM Bafilomycin A1 for 4 hours. Scale bar, 10 μm.

Appendix Figure S3*:*

(**a**) KRIT1 KO re-expressing KRIT1 (KO+KRIT1) and KRIT1 KO MEFs were transiently transfected with the genetically-encoded ratiometric H_2O_2 sensor mt-HyPer. Where indicated, cells were treated with 100nM Torin1 for 16 hours. Time-course traces on the right show a gradual increase in the mt-HyPer ratio only in CCM1 KO cells, reverted by Torin1 treatment. H_2O_2 was added as a reference. Quantifications on the left; **p*=0.01174 (KI ctrl *vs* KO ctrl); n=26 for each conditions.

(**b**) Immunoblot analysis of phosphorylated p70 S6 Kinase (Ser 371), total p70 S6 Kinase, phosphorylated 4E-BP1 (Thr 37/46), total 4E-BP1 and actin (loading marker). KRIT1 KO re-expressing KRIT1 (KO+KRIT1) and KRIT1 KO cells were treated or not with 20mM NAC or 0.5mM Tempol for 16 hours. Results are representative of three independent experiments.

(**c**) Immunoblot analysis of p62, LC3 and actin (loading marker). KRIT1 KO re-expressing KRIT1 (KO+KRIT1) and KRIT1 KO cells were treated or not with 20mM NAC or 0.5mM Tempol for 16 hours. Results are representative of three independent experiments.

(**d**) Proliferation of vehicle- 500nM Rapamycin- or 100nM Torin1-treated KRIT1 KO endothelial cells. Data are mean ± s.e.m of three independent experiments.

(**e**) Proliferation of vehicle- or Torin1-treated KRIT1 KO re-expressing KRIT1 (KO+KRIT1) and KRIT1 KO MEFs. Torin1 was used at a concentration of 100nM. Data are mean ± s.e.m of three independent experiments.

Appendix Figure S4:

(**a**) Immunoblot analysis of p62, actin, LC3I/II in HUVEC transfected negative siRNA (ctrl siRNA) or *ATG7* siRNA, for 72 hours. Results are representative of three independent experiments.

(**b**) Cell migration (expressed as percentage) of HUVEC transfected with negative siRNA (ctrl siRNA) or *ATG7* siRNA, for 72 hours. Results are representative of three independent experiments, performed in triplicate. *p=0.01477

(**c**) Cell migration (expressed as percentage) of WT and KRIT1 KO endothelial cells. Results are representative of three independent experiments, performed in triplicate. *p=2.98e^{-6}

(**d**) Cell migration (expressed as percentage) of KRIT1 KO endothelial cells treated with vehicle (KO ctrl), 100nM Torin1 (KO+Torin1) or 500nM Rapamycin (KO+Rapa) for 24 hours. Representative DAPI-fluorescent (20x magnification; scale bar: 10 µm) images have been reported. Results are representative of three independent experiments, performed in triplicate. *p=3.25e^{-23} (ctrl *vs* Torin1); *p=2.65e^{-7} (ctrl *vs* Rapa).

(**e**) Gene expression levels of EndMt markers in *p62* and control short interfering RNA (siRNA) in CCM1 KO endothelial cells. Fold changes in *p62* siRNA versus control.

(f) Immunoblots analysis with antibodies specific to phosphorylated mTOR (Ser 2448), total mTOR and total ULK1 in CCM3 WT and CCM3 KO endothelial cells; actin was used as loading marker. Results are representative of three independent experiments.

(g) 3D reconstruction of *CCM3-ECKO* merged images showed in Figure 3f, obtained with confocal Z-stack, shown in orthogonal view. Scale bar: 100 µm.

Appendix Figure S5:

(**a**) Immunoblot analysis of CCM2 and actin in EA.hy926 transfected negative siRNA (ctrl siRNA) or *CCM2* siRNA, for 72 hours. Results are representative of three independent experiments.

(**b**) Immunoblot analysis of p62, actin and LC3I/II in EA.hy926 transfected negative siRNA (ctrl siRNA) or *CCM2* siRNA, for 72 hours. Results are representative of three independent experiments.

(**c**) Immunofluorescence analysis of p62 (green) and ProteoStat Aggresome staining detection reagent (red) in EA.hy926 cells, transfected with negative siRNA (ctrl siRNA) or *CCM2* siRNA. The yellow signal in the merged images represents an overlapping spatial relationship between green and red fluorescence. Magnification in insets. Scale bar, 20 μm. The images are representative of three independent experiments.

(**d**) Immunoblot analysis of phosphorylated p70 S6 Kinase (Ser 371), total p70 S6 Kinase, phosphorylated 4E-BP1 (Thr 37/46), total 4E-BP1 and actin (loading marker) in EA.hy926 transfected with negative siRNA (ctrl siRNA) or *CCM2* siRNA. Results are representative of three independent experiments.

(**e**) Immunoblot analysis of phosphorylated ULK1 (Ser 757), total ULK1 and actin (loading marker) in EA.hy926 transfected with negative siRNA (ctrl siRNA) or *CCM2* siRNA. Results are representative of three independent experiments.
