# Supplementary material for: Defective autophagy is a key feature of cerebral cavernous malformations
Source: EMBO Mol Med. 2015 Sep 28;7(11):1403–17. doi: 10.15252/emmm.201505316 (PMC4644374; doi:10.15252/emmm.201505316)
Supplement: Supplementary file 2 [file emmm0007-1403-sd2.pdf]

EMBO Molecular Medicine

# Defective autophagy is a key feature of cerebral cavernous malformations

Saverio Marchi, Mariangela Corricelli, Eliana Trapani, Luca Bravi, Alessandra Pittaro, Simona Delle Monache, Letizia Ferroni, Simone Patergnani, Sonia Missiroli, Luca Goitre, Lorenza Trabalzini, Alessandro Rimessi, Carlotta Giorgi, Barbara Zavan, Paola Cassoni, Elisabetta Dejana, Saverio Francesco Retta and Paolo Pinton

*Corresponding author: Paolo Pinton, University of Ferrara*

**Review timeline:**

| | |
|---|---|
| Submission date: | 07 April 2015 |
| Editorial Decision: | 07 May 2015 |
| Revision received: | 06 August 2015 |
| Editorial Decision: | 20 August 2015 |
| Accepted: | 01 September 2015 |

## Transaction Report:

(Note: With the exception of the correction of typographical or spelling errors that could be a source of ambiguity, letters and reports are not edited. The original formatting of letters and referee reports may not be reflected in this compilation.)

*Editor: Céline Carret*

1st Editorial Decision 07 May 2015

Thank you for the submission of your manuscript to EMBO Molecular Medicine. We have now heard back from the two referees whom we asked to evaluate your manuscript.

You will see that both referees find the data important and are rather supportive of publication. They nevertheless have a few suggestions to improve the paper impact even more by performing additional experiments that would strengthen the findings and provide mechanistic insights.

I will not get into experimental details, but we feel that the referees' reports are very clear and nicely detailed and we would strongly encourage you to address all issues raised as recommended.

Given these evaluations, I would like to give you the opportunity to revise your manuscript, with the understanding that the referees' concerns must be fully addressed and that acceptance of the manuscript would entail a second round of review. Please note that it is EMBO Molecular Medicine policy to allow only a single round of revision and that, as acceptance or rejection of the manuscript will depend on another round of review, your responses should be as complete as possible.

EMBO Molecular Medicine has a "scooping protection" policy, whereby similar findings that are published by others during review or revision are not a criterion for rejection. Should you decide to submit a revised version, I do ask that you get in touch after three months if you have not completed it, to update us on the status.

Please also contact us as soon as possible if similar work is published elsewhere. If other work is

published we may not be able to extend the revision period beyond three months.

I look forward to receiving your revised manuscript.

***** Reviewer's comments *****

Referee #1 (Remarks):

Summary: The manuscript by Marchi et al focuses on the regulation of autophagy in the disease cerebral cavernous malformation (CCM). The authors show an accumulation of the autophagy marker p62, indicative of inhibition of autophagy, in CCM patient samples, and in genetically CCM1 deficient endothelial cells. They also investigate the role of the mTOR signaling pathway in regulation of autophagy in CCM1 deficient cells, describe a link between autophagy and endoMT, and suggest that inhibition of mTOR may counteract some of the abnormal signaling initiated by loss of CCM proteins.

Criticism:
This manuscript provides evidence that in CCM lesions and KRIT1 deficient cells, autophagy is inhibited. While there are a few issues as noted, in general the conclusions are well supported. This is a key new piece of information that will be of great interest to the field, and suggests ways that the CCM proteins (including KRIT1) could regulate endothelial behavior in normal tissues. Further interest would be generated if the authors were to determine how loss of one or more of the CCM family of proteins caused accumulation of p62 or activation of mTOR.

Major issues:

1. The use of multiple KRIT1 deficient cell lines introduces some variability in the data not suggested by the author's main conclusions. These should be addressed experimentally or by softening the language. For example, in Fig. 2, in CCM1 KO endos p62 is significantly up-regulated, however in BMVEC and in EA.hy926 cells, this up-regulation is very slight. The authors counter this by saying that total LC3 is increased, but increased expression of this protein is not a marker of autophagy. It would be better to support this data with aggresome staining in these cell lines, similar to the data shown in Fig 2 g/h. In Fig 3C, the ratio of LC3II to LC3I is increased in the presence of Torin in both Wt and CCM1 Ko cells, but rapamycin only increases LC3II in wt cells. The same can be seen in KO MEFs. Given the variance in blotting of these proteins, it is suggested that either a more careful analysis be presented, or the conclusion that the CCM pathway regulates autophagy identically in all cell types be clarified.
2. The molecular mechanisms by which loss of CCM proteins lead to the wide variety of published effects remain unclear. It is important that these effects be put into context of the current body of knowledge. In supplemental data, the authors demonstrate that the accumulation of p62 appears independent of the CCM-deficiency driven accumulation of ROS. However, there are other pathways affected by loss of CCM proteins. For example, the CHX data suggest some transcriptional dependence, does loss of CCM1 affect p62 mRNA? P62 transcription is known to be downstream of JNK, which is also activated downstream of loss of CCM1, is there a connection?
3. Figure 4 reports that loss of ATG7, which inhibits autophagy, down regulates endothelial markers and up regulates mesenchymal markers, in addition to inhibiting tube formation. The authors go on to show that blocking mTOR in CCM1 ko cells reduces expression of mesenchymal markers, but not that blocking mTOR restores endothelial markers in CCM1 ko cells. However, the heading for this section of the results is: Suppression of autophagy contributes to the pathogenesis of cerebral cavernous malformations! There is no direct evidence that suppression of autophagy contributes to CCM formation in vivo. As the contribution of EndoMT to CCM lesion formation remains controversial, this conclusion is premature and should be removed/reworded. Alternatively, substantial new evidence would be required to support this conclusion.

Minor issues:

1. The correct genetic and protein nomenclature for CCM1 is KRIT1 (for ref. see HGNC, Entrez, UniProt, OMIM, NCBI, Ensembl). The authors should refer to KRIT1 by its official name in the

manuscript to avoid confusion.
2. In supplemental table 1 and the text, the authors refer to the patient samples as sporadic, yet the majority of the samples have multiple lesions, which are considered a strong marker of hereditary CCM. The authors should indicate which of the patient samples have been genotyped, and if so, whether they are carriers of mutations in KRIT1, CCM2, CCM3, or none.
3. It would be helpful in the interpretation of the IHC images in Fig 1, particularly in b/c to include arrowheads/arrows indicating the staining being described in the text. There appears to be significant staining of cellular debris or ?? in tissue areas surrounding the lesion. A brief description of those areas, or the reason for the background staining, would also be beneficial.
4. In figure 3, total mTOR appears to be increased in CCM1 KO ec and CCM1 KO MEFs. This likely impacts the amount of "active" mTOR, but is not addressed in the analysis.

Referee #2 (Remarks):

The authors describe that autophagy is impaired upon CCM1 or CCM3 mutations in different cell lines, and most notably in human samples of cerebral cavernous malformations. This could indeed become a landmark study for the better understanding of this disease. It puts autophagy defects to the list of potential causes of cerebral cavernomas (impaired endothelial cell junctions, angiogenesis, ROS, endothelial to mesenchymal transition). The authors show how Ccm1 deficient endothelial cells have impaired autophagy that is accompanied by an overactivation of mTOR signaling pathway. Moreover inhibition of mTOR recovered partially autophagy. In general the techniques used are good and the methods are adequate, however some issues remain to be clarified in order to better support their hypothesis.
1) The first figure is more a probe of concept figure rather than a first result for the study. For this reason it should not be the first result to mention but more likely the last. The figure lacks legends and scale.
2) The reason why authors perform some of their experiments in MEF cells considering that this is an endothelial cell dependent disease should be outlined.
3) The authors should include CCM2 in their study. This would help to understand if defective autophagy is a general aspect of CCMs.
4) The authors use Torin without explaining its mechanism of action, or at least its molecular targets. Since there are some differences to Rapamycin in the efficiency to inhibit either mTORC1 (for Rapamycin) or both complexes (for Torin) the relevance of these differences should be addressed. Torin seems to have a greater effect than Rapamycin what would indicate that the effects seen are more mTORC2 dependent, what would be quite interesting. Considering how important mTORC2 is for Akt activation, the role of Akt in the process should also be addressed. Akt has a role on cell survival and proliferation, and could also be responsible of some of the effects observed. Authors mistook some of the figure references (i.e. Suppl figure 3b reference on page 7 is clearly referring to suppl figure 2b)
5) The authors state that "reduced ULK1 expression in CCM1-KO endothelial cells is strictly dependent by higher mTOR activity". However ULK1 levels are still lower than WT after mTOR pathway complete inhibition (according to p70S6K blot) so there is clearly something else lowering down ULK1 expression, and for this reason this sentence is overestimating the results.
6) The authors should explain the reason why they use any inhibitor for the reader to follow. Why Xestospongin B?
7) The method of LC3-tandem experiment is not properly explained and the paragraph is confusing. It should be explained what the different outcomes of this experiments mean in order to make it more understandable. It is not clear whether autophagosome or autolysosome accumulation is a symptom of autophagy disorder or not. The way it is explained makes it really difficult to interpret.
8) It would be interesting to evaluate if ROS inhibition leads to a decrease of mTOR activity, and to a recovery of autophagy, given that authors state that it is mTOR over-activation what is leading to an increase in ROS production and not vice versa.
9) It is demonstrated that defective autophagy leads to EndMT however the markers used to measure this EndMT are not properly explained for the reader to be able to judge if the regulation of these genes are relevant or not. Considering that the blots presented for VE-Cadherin, CD31 and alpha-SMA do not show dramatic differences. Specially because tube formation experiment could simply

mean that autophagy is necessary for tube formation, and not that it is attributable to EndMT. How are these genes in CCM1-KO cells given that authors only present ATG7 silencing experiments, and if autophagy deficiency is responsible of EndMT, then CCM1-KO cells should have mesenchymal markers too. On top of it mTOR inhibition should revert this EndMT also.
10) In the last set of results the authors decide to analyze if why other gene involved in these disorders autophagy deficiency was also observed in order to demonstrate that autophagy disorder is a common feature for CCM, and that its regulation should be considered as a therapeutic target, however authors seem to neglect the recent paper published on line in February in Aging cell in which Guerrero et al. demonstrate that CCM3 deficiency leads to a defective autophagy. This publication is recent and for that it is understandable that authors didn't cite it, however, it should be included in a potential revision of this paper.

1st Revision - authors' response 06 August 2015

**Responses to Reviewer #1**

Re: Ms. ID EMM-2015-05316 ("Defective autophagy is a key feature of cerebral cavernous malformations" by Saverio Marchi *et al.*)

*Reviewer's comments:*

> *GENERAL - "This manuscript provides evidence that in CCM lesions and KRIT1 deficient cells, autophagy is inhibited. While there are a few issues as noted, in general the conclusions are well supported. This is a key new piece of information that will be of great interest to the field, and suggests ways that the CCM proteins (including KRIT1) could regulate endothelial behavior in normal tissues. Further interest would be generated if the authors were to determine how loss of one or more of the CCM family of proteins caused accumulation of p62 or activation of mTOR."*

The authors would like to thank Reviewer #1 for his gratifying general comments. Specifically, they were very pleased to read that the reported research data and conclusions were considered well supported and a key new piece of information of great interest to the field.

Furthermore, they agree that the understanding of how loss of one or more of the CCM family of proteins causes accumulation of p62 or activation of mTOR would generate further interest. Nevertheless, identification and characterization of the underlying molecular mechanisms will require more specifically and deeply focused investigations. Indeed, whereas CCM proteins have been involved in the regulation of multiple signaling molecules and mechanisms, the central role of p62 and mTOR in the modulation of autophagy makes them a target for regulation by multiple pathways, providing a rational frame for future studies. In addition, our findings provide also a novel framework for better addressing the role of stress factors putatively related to CCM disease onset and progression, including oxidative stress and inflammation.

The major issues raised have been addressed point-by-point as follows:

*1. Reviewer's comment:*

*The use of multiple KRIT1 deficient cell lines introduces some variability in the data not suggested by the author's main conclusions. These should be addressed experimentally or by softening the language. For example, in Fig. 2, in CCM1 KO endos p62 is significantly up-regulated, however in BMVEC and in EA.hy926 cells, this up-regulation is very slight. The authors counter this by saying that total LC3 is increased, but increased expression of this protein is not a marker of autophagy. It would be better to support this data with aggresome staining in these cell lines, similar to the data shown in Fig 2 g/h. In Fig 3C, the ratio of LC3II to LC3I is increased in the presence of Torin in*

*both Wt and CCM1 Ko cells, but rapamycin only increases LC3II in wt cells. The same can be seen in KO MEFs. Given the variance in blotting of these proteins, it is suggested that either a more careful analysis be presented, or the conclusion that the CCM pathway regulates autophagy identically in all cell types be clarified.*

1. Authors' reply:

We agree with the reviewer that, based on the evidence available in the original manuscript, we had overstated in claiming that CCM proteins regulate autophagy identically in all cell types. As suggested, we have supported the data obtained in hBMECs and EA.hy 926 cells with immunostaining for p62 and aggresomes (Supplementary Fig. 1f-g); these results correlate with the observations made in KRIT1-KO cells. However, the increase in p62 levels in human endothelial cells is lower than that in murine KO models. This aspect could be reasonably due to the cellular stress response induced by transient transfections (performed for 48 or 72 h), which subsequently led to activation of the autophagic process, as evidenced by the high basal LC3-II/I ratio under control conditions (i.e., transfected with negative siRNAs; see Fig. 1e-f and Supp. Fig. 5b). Thus, the unavoidable transfection-dependent induction of autophagy could partially mask the effects of specific *CCM* silencing.

We stated that rapamycin is able to re-activate autophagy even in KO cells based on the lower levels of p62 and increased LC3-II/I ratio compared to untreated conditions. However, rapamycin is less efficient than Torin1 in stimulating autophagy and in reverting some pathological features of KO cells. Consistently, Torin1 has been reported to be more effective than rapamycin in inhibiting mTORC1 (PMID: 24913553), as well as to activate autophagy to a greater extent than rapamycin independently of its putative action on mTORC2 (PMID: 19395872). Therefore, the higher efficacy of Torin1 treatment to drive autophagy is likely attributable to its greater effect on autophagy, as compared with rapamycin. These considerations, which were missing in the previous version of the manuscript, have been now included in the revised version along with appropriate references (page 7).

According to the reviewer's comments and suggestions, the additional experimental data and information included in the revised version of the manuscript should better support the experimental findings and conclusions.

*2. Reviewer's comment:*

*The molecular mechanisms by which loss of CCM proteins lead to the wide variety of published effects remain unclear. It is important that these effects be put into context of the current body of knowledge. In supplemental data, the authors demonstrate that the accumulation of p62 appears independent of the CCM-deficiency driven accumulation of ROS. However, there are other pathways affected by loss of CCM proteins. For example, the CHX data suggest some transcriptional dependence, does loss of CCM1 affect p62 mRNA? P62 transcription is known to be downstream of JNK, which is also activated downstream of loss of CCM1, is there a connection?*

2. Authors' reply:

We thank the reviewer for its useful suggestions. Indeed, loss of CCM proteins has potentially pleiotropic effects on numerous biological pathways. Interestingly, most of the reported effects, including the induction of c-Jun (Goitre et al., 2004) and b-catenin (Bravi et al., 2015) signaling, and endothelial-to-mesenchymal transition (Maddaluno et al., 2013), are known to be either upstream or downstream of autophagic dysfunctions (PMID: 20404571 and 25633614, and this paper), suggesting that autophagy may play a pivotal role in mediating CCM protein functions.

As mentioned by the reviewer, whereas p62 is regulated at transcriptional level by the JNK pathway, JNK is activated downstream of loss of *CCM1* (Goitre et al., 2014), suggesting a potential connection. This relevant information has now been considered in the revised version of the MS. However, by measuring p62 mRNA levels in wt and KRIT1-KO endothelial cells, we detected no significant differences (Supplementary Fig. 1c), suggesting that p62 accumulation observed in KRIT1-KO cells could be primarily due to autophagy suppression. Nevertheless, we cannot totally

exclude the possibility that p62 expression could be additionally regulated to some extent also at the transcriptional level, particularly under conditions that induce higher ROS levels and ROS-dependent activation of JNK.

*3. Reviewer's comment:*

*Figure 4 reports that loss of ATG7, which inhibits autophagy, down regulates endothelial markers and up regulates mesenchymal markers, in addition to inhibiting tube formation. The authors go on to show that blocking mTOR in CCM1 ko cells reduces expression of mesenchymal markers, but not that blocking mTOR restores endothelial markers in CCM1 ko cells. However, the heading for this section of the results is: Suppression of autophagy contributes to the pathogenesis of cerebral cavernous malformations! There is no direct evidence that suppression of autophagy contributes to CCM formation in vivo. As the contribution of EndoMT to CCM lesion formation remains controversial, this conclusion is premature and should be removed/reworded. Alternatively, substantial new evidence would be required to support this conclusion.*

3. Authors' reply:

We agree with the reviewer that previous conclusions related to Figure 3 (Fig. 4 in the previous version) were not fully supported by experimental data. According to the reviewer's suggestions, we reworded the entire section and softened the language throughout the text, including the headings for this section and Fig. 3 legend, which now state as follow: Defective autophagy underlies major phenotypic signatures of CCM disease. Furthermore, we provided new experimental evidence to reinforce the correlation between EndMt and defective autophagy. In particular, we show that mTOR inhibition restores endothelial markers in KRIT1-KO cells (Fig. 3b). Moreover, ATG7-silencing in HUVECs slowed the formation of capillary-like structures (Fig. 3d) but significantly increased the migratory capacity of these cells (Supplementary Fig. 4b). Importantly, inhibition of mTOR signaling reduced the migration of KRIT1-KO endothelial cells (Supplementary Fig. 4c-d). These data are consistent with recent observations (Singh et al, 2015; Zhang et al, 2013), translating the association between mTOR-dependent inhibition of autophagy and EndMt to CCM disease. In addition, we demonstrate that defective autophagy and consequent p62 accumulation are common features of loss-of-function mutations of all three known CCM genes. These novel results are presented and discussed on pages 8-9.

The minor issues raised have been addressed point-by-point as follows:

*1. Reviewer's comment:*

*The correct genetic and protein nomenclature for CCM1 is KRIT1 (for ref. see HGNC, Entrez, UniProt, OMIM, NCBI, Ensembl). The authors should refer to KRIT1 by its official name in the manuscript to avoid confusion.*

1. Authors' reply:

According to the reviewer's comment, the genetic and protein nomenclature for *CCM1* has been corrected to *KRIT1* in the revised version of the manuscript.

*2. Reviewer's comment:*

*In supplemental table 1 and the text, the authors refer to the patient samples as sporadic, yet the majority of the samples have multiple lesions, which are considered a strong marker of hereditary CCM. The authors should indicate which of the patient samples have been genotyped, and if so, whether they are carriers of mutations in KRIT1, CCM2, CCM3, or none.*

2. Authors' reply:

Histological samples of human CCM lesions were obtained from paraffin-embedded surgically resected CCM specimens retrieved from the archives of the Department of Anatomy and Diagnostic Histopathology at the "Città della Salute e della Scienza" University Hospital, Turin, Italy. Indeed, as correctly noticed by the reviewer, most of the selected CCM specimens were linked to patients carrying multiple CCM lesions, which is considered indicative of the familial (inherited) form of the disease, whereas some specimens were derived from carriers of a single CCM lesion, which might be suggestive of a sporadic case. Nevertheless, neither documented family history information nor genetic data were available from most of the corresponding medical records; thus, the precise nature of the selected CCM cases remains undefined. Consequently and according to the reviewer's comment, we removed any specific reference to either sporadic or familial cases in the revised version of the manuscript, referring more generally to CCM lesions. Notably, compared to normal vasculature in peri-lesional areas, p62 expression was enhanced in CCM vessels of surgical specimens from either single or multiple CCM lesion carriers, suggesting a general phenomenon.

A more detailed description of the origin of the CCM specimens used in our histological and immunohistochemical studies has been added in the Materials and Methods (page 13) and Results (page 10) sections of the revised version of the manuscript.

*3. Reviewer's comment:*

*It would be helpful in the interpretation of the IHC images in Fig 1, particularly in b/c to include arrowheads/arrows indicating the staining being described in the text. There appears to be significant staining of cellular debris or ?? in tissue areas surrounding the lesion. A brief description of those areas, or the reason for the background staining, would also be beneficial.*

3. Authors' reply:

According to the reviewer's comment, arrows have been added in the revised version of the manuscript to facilitate the interpretation of the IHC images in Figure 1 (Fig. 4 in the revised version of the manuscript), indicating the staining being described in the text.

In particular, arrows show cerebral vessel endothelial cells either positive or negative for p62 staining, whereas the background staining in brain parenchyma surrounding CCM lesions marks either cell debris or p62 immunoreactivity in neuronal and glial cells.

*4. Reviewer's comment:*

I*n figure 3, total mTOR appears to be increased in CCM1 KO ec and CCM1 KO MEFs. This likely impacts the amount of "active" mTOR, but is not addressed in the analysis.*

4. Authors' reply:

We thank the reviewer for this suggestion. This observation has been included and discussed in the current MS (page 7).

**Responses to Reviewer #2**

Re: Ms. ID EMM-2015-05316 ("Defective autophagy is a key feature of cerebral cavernous malformations" by Saverio Marchi et al.)

*Reviewer's comments:*

> *GENERAL - "The authors describe that autophagy is impaired upon CCM1 or CCM3 mutations in different cell lines, and most notably in human samples of cerebral cavernous malformations. This could indeed become a landmark study for the better understanding of this disease. It puts autophagy defects to the list of potential causes of cerebral cavernomas (impaired endothelial cell junctions, angiogenesis, ROS, endothelial to mesenchymal transition). The authors show how Ccm1 deficient endothelial cells have impaired autophagy that is accompanied by an overactivation of mTOR signaling pathway. Moreover inhibition of mTOR recovered partially autophagy. In general the techniques used are good and the methods are adequate, however some issues remain to be clarified in order to better support their hypothesis."*

The authors would like to thank Reviewer #2 for his gratifying and useful comments. In particular, the authors were very pleased to read that their study could become a landmark study for the better understanding of CCM disease, putting autophagy defects to the list of potential causes of cerebral cavernomas (impaired endothelial cell junctions, angiogenesis, ROS, endothelial to mesenchymal transition).

The issues raised have been addressed point-by-point as follows:

*1. Reviewer's comment:*

*The first figure is more a probe of concept figure rather than a first result for the study. For this reason it should not be the first result to mention but more likely the last. The figure lacks legends and scale.*

1. Authors' reply:

According to the reviewer's suggestion, we agreed to consider the first figure as a probe of concept figure rather than a first result for the study. Therefore, we moved it to the end (now Fig. 4) and discussed the results accordingly. Moreover, the relative figure legend has been improved and scale included.

*2. Reviewer's comment:*

*The reason why authors perform some of their experiments in MEF cells considering that this is an endothelial cell dependent disease should be outlined.*

2. Authors' reply:

The reason why some experiments were exclusively performed in MEFs, rather than endothelial cells, resides in the investigation of the relationship between ROS and autophagy. Indeed, KRIT1-KO endothelial cells displayed a slight increase in ROS levels compared to their counterpart KO MEFs likely because endothelial cells possess a more efficient ROS-scavenging system than a fibroblast-like cellular model. Therefore, the putative role of ROS in the transcriptional regulation of p62 levels (Supp. Fig. 1a-b) and the effects of mTOR inhibition on ROS production (Supp. Fig. 3a-c) have been analyzed only in MEFs. However, in the previous version of the paper, the amounts of

p62 in the TX-100 soluble and TX-100 insoluble fractions were erroneously analyzed only in MEFs. The analogous experiment has now been conducted in endothelial cells (Supp. Fig. 1d).

*3. Reviewer's comment:*

*The authors should include CCM2 in their study. This would help to understand if defective autophagy is a general aspect of CCMs.*

3. Authors' reply:

We thank the reviewer for this suggestion. We reported the experimental findings regarding CCM2 down-regulation and autophagy in Supplementary Fig. 5. These novel results have strongly reinforced the primary conclusions of the manuscript, demonstrating that defective autophagy is a common feature of loss-of-function mutations of all three known CCM genes.

*4. Reviewer's comment:*

*The authors use Torin without explaining its mechanism of action, or at least its molecular targets. Since there are some differences to Rapamycin in the efficiency to inhibit either mTORC1 (for Rapamycin) or both complexes (for Torin) the relevance of these differences should be addressed. Torin seems to have a greater effect than Rapamycin what would indicate that the effects seen are more mTORC2 dependent, what would be quite interesting. Considering how important mTORC2 is for Akt activation, the role of Akt in the process should also be addressed. Akt has a role on cell survival and proliferation, and could also be responsible of some of the effects observed. Authors mistook some of the figure references (i.e. Suppl figure 3b reference on page 7 is clearly referring to suppl figure 2b).*

4. Authors' reply:

According to the reviewer's suggestion, the mechanisms of action of Rapamycin and Torin 1, respectively an allosteric and a small molecule ATP-competitive inhibitor of mTOR, have been now reported in the revised version of the MS. Indeed, Rapamycin is less efficient than Torin1 in stimulating autophagy and reverting some pathological features of KO cells. Consistently, Torin1 has been reported to be more effective than Rapamycin in inhibiting mTORC1 (PMID: 24913553), as well as to activate autophagy to a greater extent than Rapamycin independently of its putative action on mTORC2 (PMID: 19395872). Specifically, Torin1 has been show to induce autophagy to the same extent in both wt cells and cells lacking Rictor, a required mTORC2 component (PMID: 19150980; PMID: 19395872). Therefore, the higher efficacy of Torin1 treatment to drive autophagy is likely attributable to its greater effect on autophagy, as compared with Rapamycin (see also "response to reviewer #1", query 1). These considerations, which were missing in the previous version of the manuscript, have been now included in the revised version along with appropriate references (page 7).

In the light of its main functional interaction with mTORC2, it is tempting to speculate that Akt is not a major candidate player in molecular mechanisms linking CCM proteins to autophagy regulation. Nevertheless, we agree that addressing its putative role would be relevant in order to understand how loss-of-function of CCM proteins causes accumulation of p62 and activation of mTOR, thus providing additional information and generating further interest on the reported findings. However, it would require further and more specific investigations, which we would like to undertake in future specifically and deeply focused studies along with the analysis of other putative candidates in the attempt to better characterize the molecular mechanisms underlying the identified relationship between loss-of-function of CCM proteins and defective autophagy.

According to the reviewer's comment, the indicated figure references have been corrected.

*5. Reviewer's comment:*

*The authors state that "reduced ULK1 expression in CCM1-KO endothelial cells is strictly dependent by higher mTOR activity". However ULK1 levels are still lower than WT after mTOR pathway complete inhibition (according to p70S6K blot) so there is clearly something else lowering down ULK1 expression, and for this reason this sentence is overestimating the results.*

5. Authors' reply:

We agree with the reviewer that, based on the evidence available in the original manuscript, we had overstated in claiming that reduced ULK1 expression is strictly dependent on mTOR activity. Thus, we have softened the language of the relative section and attempted to reinforce our conclusions through the analysis of phospho-AMBRA1 levels. Indeed, mTOR phosphorylates the autophagy regulator AMBRA1 at Ser 52, leading to ULK1 destabilization and consequent degradation (Nazio et al, Nat Cell Biol 2013). As shown in Supplementary Fig. 3a, KRIT1-KO ECs displayed higher levels of mTOR-dependent AMBRA1 phosphorylation. Thus, mTOR hyper-activation might contribute, at least partially, to the reduced ULK1 expression observed in KO cells.

*6. Reviewer's comment:*

*The authors should explain the reason why they use any inhibitor for the reader to follow. Why Xestospongin B?*

6. Authors' reply:

We used Xestospongin B as an mTOR-independent pro-autophagic stimulus. Its mechanism of action has now been reported (page 8).

*7. Reviewer's comment:*

*The method of LC3-tandem experiment is not properly explained and the paragraph is confusing. It should be explained what the different outcomes of this experiments mean in order to make it more understandable. It is not clear whether autophagosome or autolysosome accumulation is a symptom of autophagy disorder or not. The way it is explained makes it really difficult to interpret.*

7. Authors' reply:

We agree with the reviewer. The explanation of the LC3-tandem method has been enriched and ameliorated (pages 7-8).

*8. Reviewer's comment:*

*It would be interesting to evaluate if ROS inhibition leads to a decrease of mTOR activity, and to a recovery of autophagy, given that authors state that it is mTOR over-activation what is leading to an increase in ROS production and not vice versa.*

8. Authors' reply:

We want to thank the reviewer for this suggestion. We treated MEFs cells with the well-known antioxidants NAC and tempol. As shown in Supplementary Fig. 4b-c, ROS scavenging did not affect mTOR hyper-activation in KO cells and failed to restore autophagy, confirming that mTOR over-activation and consequent autophagy inhibition are upstream of ROS production. These novel results have been presented and discussed on page 8.

*9. Reviewer's comment:*

*It is demonstrated that defective autophagy leads to EndMT however the markers used to measure this EndMT are not properly explained for the reader to be able to judge if the regulation of these genes are relevant or not. Considering that the blots presented for VE-Cadherin, CD31 and alpha-SMA do not show dramatic differences. Specially because tube formation experiment could simply mean that autophagy is necessary for tube formation, and not that it is attributable to EndMT. How are these genes in CCM1-KO cells given that authors only present ATG7 silencing experiments, and if autophagy deficiency is responsible of EndMT, then CCM1-KO cells should have mesenchymal markers too. On top of it mTOR inhibition should revert this EndMT also.*

9. Authors' reply:

According to the reviewer's suggestion,, we ameliorated the explanation of the markers used to describe EndMt. Moreover, as shown in Figure 3a, KRIT1-KO displayed higher levels of mesenchymal markers, whereas treatment with either Torin1 or rapamycin significantly reduced their expression. Furthermore, we showed that mTOR inhibitors restored endothelial marker expression (Fig. 3b).

Then, we accompanied the tube formation assay with cell migration analysis (Supplementary Fig. 4b-d). Impaired tube formation and contemporary increased cell migration are considered typical traits of EndMt. Taken together, these new findings should provide further support to the link between defective autophagy and EndMt.

*10. Reviewer's comment:*

*In the last set of results the authors decide to analyze if why other gene involved in these disorders autophagy deficiency was also observed in order to demonstrate that autophagy disorder is a common feature for CCM, and that its regulation should be considered as a therapeutic target, however authors seem to neglect the recent paper published on line in February in Aging cell in which Guerrero et al. demonstrate that CCM3 deficiency leads to a defective autophagy. This publication is recent and for that it is understandable that authors didn't cite it, however, it should be included in a potential revision of this paper.*

10. Authors' reply:

The recent paper published in Aging Cell by Guerrero et al. has been included in the reference list of the revised version of the manuscript and mentioned in the Discussion section. Indeed, this paper is extremely important, as it helps demonstrate that autophagy disorder is a common feature for CCM.

2nd Editorial Decision 20 August 2015

---

Thank you for the submission of your revised manuscript to EMBO Molecular Medicine. We have now received the enclosed reports from the referees that were asked to re-assess it. As you will see the reviewers are now supportive and I am pleased to inform you that we will be able to accept your manuscript pending some final editorial amendments.

***** Reviewer's comments *****

Referee #1 (Remarks):

Reviewer's comments have been addressed appropriately.

Referee #2 (Remarks):

The authors have nicely addressed all of my concerns.

© EMBO
