# Supplementary figures and images for: Defective autophagy is a key feature of cerebral cavernous malformations
Source: EMBO Mol Med. 2015 Sep 28;7(11):1403–17. doi: 10.15252/emmm.201505316 (PMC4644374; doi:10.15252/emmm.201505316)

**A**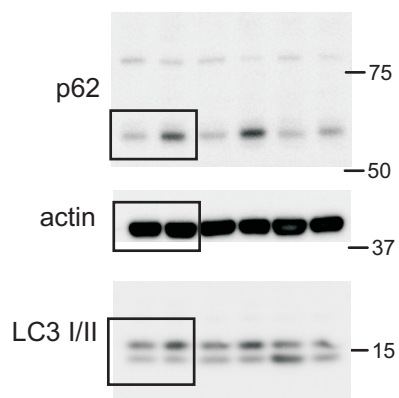**C**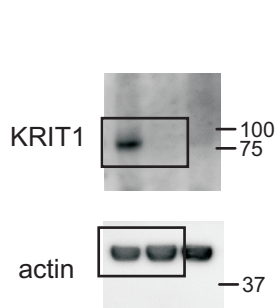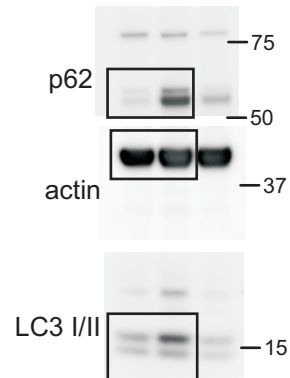**E**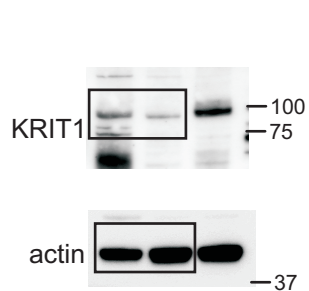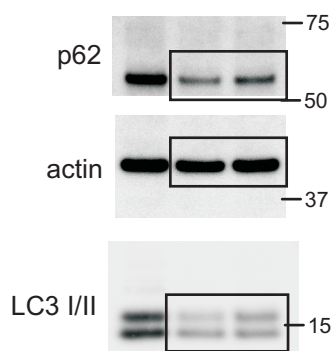**F**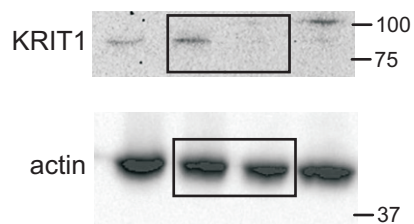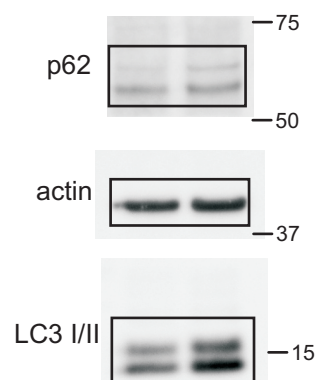

Supplement: Supplementary file 3 [file emmm0007-1403-sd3.pdf]

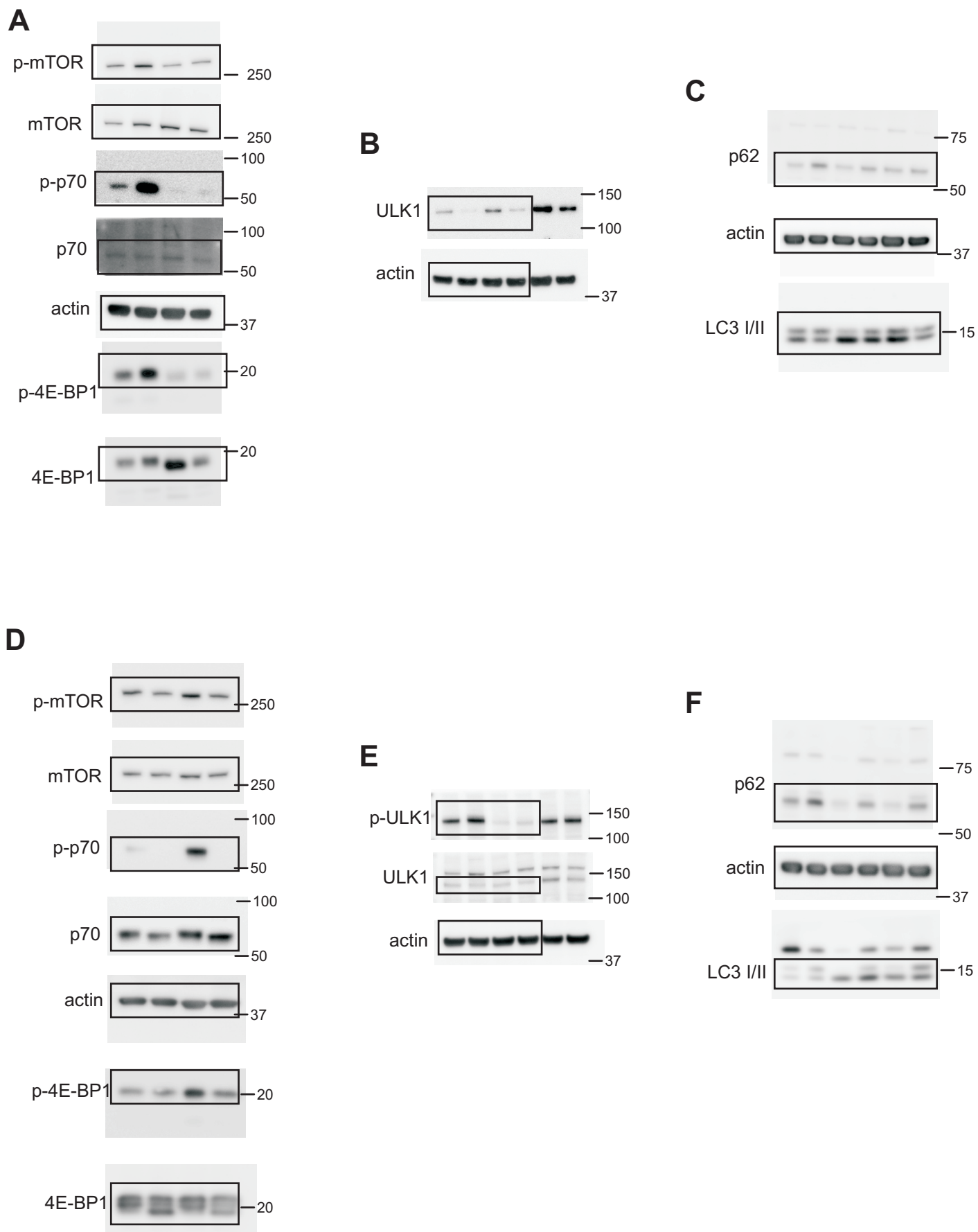

Source data for Fig.2

Supplement: Supplementary file 4 [file emmm0007-1403-sd4.pdf]

**B**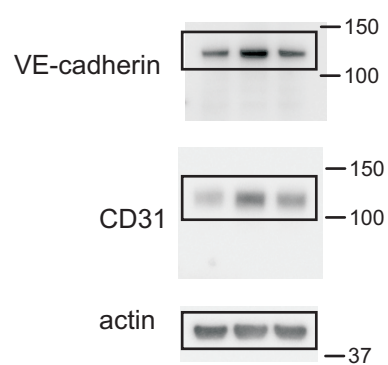**C**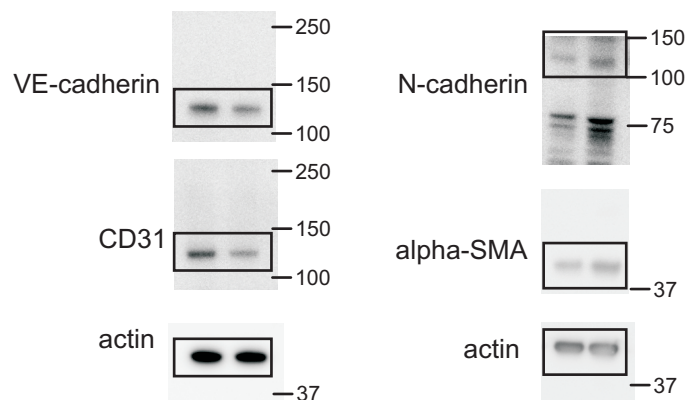**E**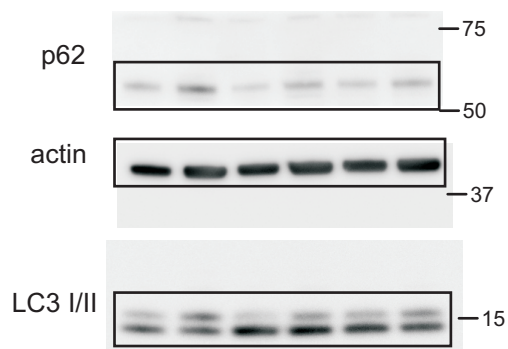

Supplement: Supplementary file 5 [file emmm0007-1403-sd5.pdf]

**A**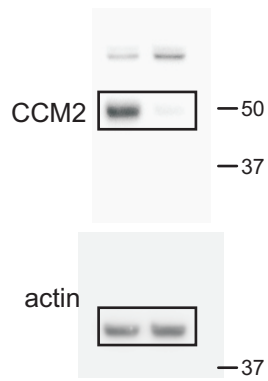**B**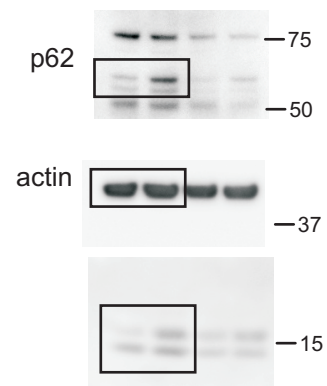**D**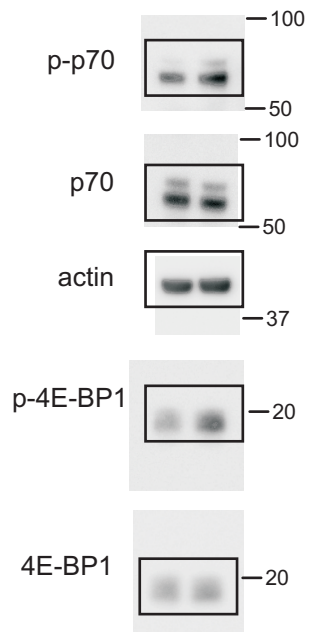**E**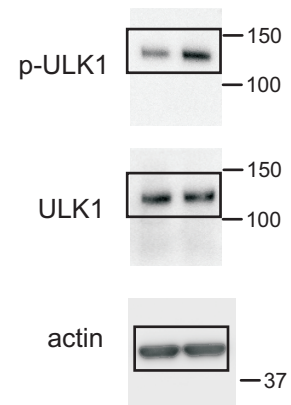

Supplement: Supplementary file 6 [file emmm0007-1403-sd6.zip › Source_data_for_Appendix.pdf]
